# Abstracts From the First Annual Research Day Hosted by the Michigan State University College of Osteopathic Medicine, Novi, Michigan, May 15, 2023.

**DOI:** 10.51894/001c.115618

**Published:** 2024-03-27

**Authors:** Andrea Amalfitano, Patricia Obando, Rana Ismail, Francis Akenami

**Affiliations:** 1 College of Osteopathic Medicine Michigan State University

**Keywords:** Michigan State University, College of Osteopathic Medicine, Novi-Michigan, First Annual Research Day in 2023, Abstracts, Spartan Medical Research Journal

## Abstract

The Spartan Medical Research Journal (SMRJ) is pleased to publish abstracts from the First Annual Research Day hosted by the Michigan State University College of Osteopathic Medicine (MSUCOM), held in Novi, Michigan, on May 15, 2023. Sponsored by MSUCOM, the Statewide Campus System (SCS), and Research, Innovation, and Scholarly Engagement (RISE), this event showcased a total of 139 selected research abstracts following a meticulous blinded review by the MSUCOM Research Day Planning Committee and SMRJ editorial staff. These abstracts were subsequently presented at the MSUCOM First Annual Research Day in 2023, with awards for exceptional oral and poster presentations conferred on May 15, 2023. Of the 139 presentations that were ultimately chosen, 45 authors consented and elected to have their abstracts published in SMRJ.

The abstracts from 2023 encompass a wide array of contemporary medical and clinical subjects, incorporating a variety of research designs that cover basic science, clinical research, case reports, medical education, and quality improvement. While abstracts offer concise overview of research projects or presentations, they do not permit a comprehensive evaluation of the scientific rigor employed in the respective works. Although these abstracts offer preliminary results that may necessitate further refinement and validation, they serve a vital function in disseminating novel research concepts and advancements in the discipline of medicine. This knowledge-sharing promotes meaningful dialogue among researchers, clinicians, and educators, thereby making a valuable contribution to the collective body of knowledge in the fields of medical sciences and osteopathic medicine. **Andrea Amalfitano, DO, PhD** Osteopathic Heritage Foundation Professor of Pediatrics, Microbiology and Molecular Genetics Professor, BioMolecular Science Gateway Editor-in-Chief, Spartan Medical Research Journal (SMRJ) MSU College of Osteopathic Medicine- Statewide Campus System **C. Patricia Obando S., PhD** Associate Dean and DIO, Graduate Medical Education Associate Professor- MSU College of Osteopathic Medicine- Statewide Campus System **Rana Ismail, PhD, MSc, CPHQ** Director of Research Editor, Spartan Medical Research Journal (SMRJ) MSU College of Osteopathic Medicine- Statewide Campus System **Francis Akenami, BMLS, PhD, MSc, FIMLS** Managing Editor Spartan Medical Research Journal (SMRJ) MSU College of Osteopathic Medicine- Statewide Campus System

## Table of Contents

**Abstracts From the First Annual Research Day Hosted by the Michigan State University College of Osteopathic Medicine, Novi, Michigan, May 15, 2023.** Andrea Amalfitano, DO, PhD, Patricia Obando, PhD, Rana Ismail, PhD, MSc, CPHQ, Francis Akenami, BMLS, PhD, MSc, FIMLS

1. **Differential Effect of Age on Mortality in African Americans Infected with the SARS-Cov-2 Virus in the United States.** Kevin Kwamin, MD (Resident); Dylan Arroyo; Blake Hardin; Hope Ring, MD; Daniel Keyes; MD, MPH (Faculty) 5
2. **Development and Evaluation of a Virtual Reality Simulation Environment for Cardiology Education: A Pilot Study.** Chris Jacob, DO (Fellow); Brilio (Jon) Mojares, MD, FACC (Faculty) 5
3. **Biochemical, Structural, and Allergenic Characterization of Plant and Mite Profilins.** Andrea O’Malley, PhD (Post-doctoral Researcher); Avery Carriuolo, BS; Sahana Sankaran, BS; Krzysztof Kowal, MD; Maksymilian Chruszcz, PhD (Faculty) 6
4. **Skin Injury Progression Following Dermal Nitrogen Mustard Exposure in C57BL/6 and Mast Cell Deficient Mice.** Andrew Roney (Trainee with Faculty); Dinesh Goswami; Ebenezar Okoyeocha; Steve Lundback; Holly Wright; Omid Madadgar; Ellen Kim; Jared M Brown; Neera Tewari-Singh (Faculty) 6
5. **Mimicking Gender-affirming Treatment of Pubertal Suppression Followed by Testosterone on Bone Growth in Female-born Mice.** Brandon W. Henry (Medical Student), Cynthia Dela Cruz PhD (Post-doc Fellow); Robert W. Goulet, PhD; Bonnie T. Nolan; Vasantha Padmanabhan, PhD; Molly Moravek, MD; Ariella Shikanov, PhD, FACP; Megan L. Killian, PhD, FACP (Faculty) 7
6. **Structural Characterization of Complex Between House Dust Mite Allergen Der p2 and human-derived IgE mAb 4C8 Fab.** Kriti Khatri, PhD (Professional Aide), Alyssa Ball, PhD; Jill Glesner; Anna Pomés, PhD; Scott Alan Smith, PhD, MD; Maksymilian Chruszcz, PhD (Faculty) 7
7. **Polyunsaturated Fatty Acid Metabolism Involvement in Age-Associated Neurodegeneration.** Jennifer Hinman (PhD Student); Morteza Sarparast (Graduate Student); Elham Pourmand (Graduate Student); Jamie Alan, RPh, PharmD, PhD (Faculty); Kin Sing Stephen Lee, PhD (Faculty) 8
8. **Unravelling the specificity of BMP 9 and BMP 10 signaling via BMPR2 in SMAD 1/5/8 Activation.** Kit Yee Chu (DO/PhD student); Dr Erik Martinez- Hackert (Faculty, PI) 8
9. **Without a Trace: The Pirin and NF-kB Interaction.** Melissa Meschkewitz, BS (DO/PhD Student); Erika M Lisabeth, PhD (Faculty); Richard R Neubig, MD, PhD (Faculty) 9
10. **Development of Novel Immunotherapy Using a Viral Multi-Gene Delivery System to Treat Human Papillomavirus-Positive Head and Neck Cancer.** Nicholas S. Giacobbi, MS (DO/PhD Student); Harrison Nabors (Undergraduate Student); Shreya Mullapudi, BS (Medical Student); Dohun Pyeon, PhD (Faculty) 9
11. **Bone Benefits of Dietary Prune in Healthy Female Mice are Time Dependent and Associated with Drastic Shifts in Gut Microbiota Composition.** Nicholas J. Chargo (DO/PhD Student); Kavisha Patel; Leon Regnery; Robert A. Quinn, PhD; Narayanan Parameswaran, PhD (Faculty); Laura R. McCabe, PhD (Faculty) 10
12. **Progression of Chloropicrin-Induced Ocular Injury: Clinical and Biological Effects.** Okoyeocha OM Ebenezar (PhD Student); Andrew Roney; Dinesh G Goswami; Lauren Gizinski; Zubeida Abouarko; J Mark Petrash; Dodd Sledge; András M Komáromy; Karen Liby; Neera Tewari-Singh, PhD (Faculty) 10
13. **The Role of LPA in Glial Signaling of Visceral Pain.** Visha Parmar (DO/PhD Student); Brian Gulbransen, PhD (Faculty, PI) 11
14. **Massage-Like Stroking Produces Analgesia in Mice.** Zachary M.S. Waarala (Medical Student); Logan Comins (Medical Student); Geoffrey Laumet, Ph.D. (Faculty) 11
15. **A Complicated Case of Heparin-Induced Thrombocytopenia.** Ananda Vigneswari Anebarassou MD, MPH (Resident); Jeevana Sreeja Ambati, MD (Resident); Sujatha Kambhatla, MD (Faculty) 12
16. **A Lethal Case of Atypical Chest Pain.** Andrew Victor, MD (Resident); Hadeel Barrawi, DO (Resident); Dylan Mooney, DO (Faculty) 12
17. **Spontaneous Bacterial Peritonitis and Pneumatosis Coli in the Presence of Pasteurella multocida Infection Without Evidence of Recent Animal Bite or Scratch.** Ariela Alonso, DO (Resident); Leonie Deniau (Medical Student); Latha Kannan, MD (Faculty) 13
18. **Surrogate Markers for Toxic Alcohol Ingestion Guide Management: A Case Report of Accidental Ethylene Glycol Intoxication.** Ashish Tripathi, BS (Medical Student); Anthony Teta, MD; Christopher Provenzano, MD (Faculty) 13
19. **Persistent Heart Failure Despite Medical Therapy, Leading to a Diagnosis of Cardiac Amyloidosis.** Diva Maraj, MD (Resident); Parth Patel, MD (Resident); Sruthi Ramanan, MD (Resident); Mumtaz Memon, MD, FACP (Faculty) ; Elise Hawes, BAS, ACS, RDCS, FASE (Faculty) 14
20. **Endoscopic Cholangioscopy with Cholelithiasis Lithotripsy Using Minimally Invasive Percutaneous (MIPS) Trilogy Lithotripter: A Case Report.** Gabriela Mazur, DO (Resident); Anthony Bonzagni, DO; Harris Shaikh, DO; Shehbaz Shaikh, MD; Robert Elgin, DO (Faculty) 14
21. **Bioinductive Collagen Implant Augmentation for Myotendinous Achilles Rupture in a Teenage Competitive Gymnast: A Case Report.** Josiah Valk, DO (Resident); Michael J Wilk, DO (Resident); Kelly Murdock, PA-C (Physician Assistant); Mohamed Saad, MD (Faculty) 15
22. **A Case of Recurrent Left-Sided Hepatic Hydrothorax in a Chronically Debilitated Patient: Uncommon Presentation.** Khaled M. Harmouch, MD (Resident); Hassan M.A. Makki, MD, FCCP (Faculty) 15
23. **An Unexpected Post-Partum Complication of Hemorrhagic Stroke.** Lucy Bolerjack, DO (Resident); Sarah Hopkins, DO (Resident); Andrew Victor, MD (Resident); Jennifer DeAnna, DO (Faculty); Dylan Mooney, DO (Faculty) 16
24. **An Unusual Presentation of a Rare Phenomenon: Hemorrhagic Cholecystitis in the Setting of Unexplained Anemia.** Rachel Cohen, DO (Resident); Dillon Jarbo, MD (Resident); Devan Schlund, MD (Faculty) 16
25. **A Rare Case of Gastrointestinal Vasculitis Confirmed by Radiological Modality.** Rashona A. Moss, DO, (Resident); Shurook Elassouli, MD; Sujata Kambhatla, MD (Faculty) 17
26. **Chronic Pulmonary Aspergillosis in an Immunocompetent Patient.** Sahil Sethi (Medical Student); Ahmad Al-Sahli, MD (Resident) 17
27. **An Unusual Case of ABO Hemolytic Disease Involving Maternal Blood Type A and Neonatal Blood Type AB.** Sandeep Bhatti, DO; Prasiksha Sitaula, MD; Kanta Bhambhani, MD; Bulent Ozgonenel, MD (Faculty) 18
28. **Lip Swelling as Sole Presenting Symptom in 4-Year-Old Female Found to Have Crohn’s Disease.** Sushmitha Meghashyam, MD; Sandeep Bhatti, DO (Faculty) 18
29. **Atypical Presentation of Acute Hemorrhagic Cholecystitis.** Shamaiza Waqas, MD; Stephen Kehres, DO (Supervising Faculty); Waqas Abid, MD; Matthew Stander, DO (Faculty) 19
30. **Bladder Cancer Causing Bladder Perforation, Resulting in Emergent Cystoprostectomy With Ileal Loop Diversion.** Upahvan Rai, DO; Brandon Tompkins, DO (Resident); Seth Thomas, DO; Ali Rizvi, BS; Richard Sarle, MD (Faculty) 19
31. **Texture Analysis of Femoral Cartilage Ultrasound Highlights Subtle Between Limb Differences in Cartilage Health in Patients After ACL Reconstruction.** Anthony L. Lewis (Medical student); Matthew S. Harkey, PhD, ATC (Faculty) 20
32. **Hospital Outcomes for ICU Patients with Sputum Positive Severe Community-Acquired Pneumonia.** Brandon Govan, MD (Resident) 20
33. **The Enigma of Eosinophilic Esophagitis and Peripheral Eosinophilia.** Brandon Wiggins, DO, MPH (Resident); Reginald Sandy, DO (Faculty) 21
34. **The Association of Hidradenitis Suppurativa with Type 2 Diabetes Mellitus in Black/African American Patients.** Eglal Samir, BS (Medical Student); Andrea Dai, BS; Rachel Krevh, BS; Ian Loveless, MS; Wan-Ting Su, PhD; Li Zhou, MD; Indra Adrianto, PhD; Qing-Sheng Mi, MD, PhD (Faculty) 22
35. **Arterial Stiffness Assessed Using Carotid-to-Femoral Pulse Wave Velocity is Unchanged Throughout the Day in Young Healthy Adults.** Hemal Dholakia (medical student); Jill M. Slade, PhD (Faculty); Katharine D. Currie, PhD (Faculty) 22
36. **Inter-Arm Blood Pressure Differences in Post-Menopausal Females with Hypertension.** Hesham Atoui (Medical Student); Emily Ubbens (Medical Student); Kasandra Sanidad (Medical Student); Wesley T. Blumenburg, MS ; Marianne Huebner, PhD; Supratik Rayamajhi, MD; George S. Abela, MD; Jill M. Slade, PhD (Faculty); Katharine D. Currie, PhD (Faculty) 23
37. **Does Early Administration of Tranexamic Acid Decrease Perioperative Blood Loss and Transfusion Rates in Addition to Intraoperative Tranexamic Acid for Hip Fracture Patients?** Jake Stahnke, DO (Resident); Shayan Memar (Medical Student); Amy Meyers, DO (Resident); Matthew Marquart D.O., FAAOS (Faculty); Jacob Lytle, DO (Fellow); Jacob Hinkley, DO, MS (Fellow) 23
38. **A Pilot Longitudinal Fixel-Based Analysis of Diffusion Weighted Magnetic Resonance Imaging Data: A TRACK TBI Study.** Joshua H. Baker, MS (Medical Student); Andrew Bender, PhD; Norman Scheel, PhD; Joshua Hubert, BS; Zac Fernandez BS; David C. Zhu, PhD (Faculty); The TRACK TBI Study Investigators 24
39. **The Effects of Anterior Cruciate Ligament Reconstruction Graft Type on Patellar Tendon Thickness.** Lauren Grasso (Medical Student); Matthew Harkey, PhD (Faculty); Lee Chou (Medical Student) 24
40. **Motor Control and Learning of a Trunk Tracking Task are Similar in Individuals With and Without Chronic Low Back Pain.** Lee S. Chou, MPH (Medical Student); Nicholas E. Bray; Angela S. Lee, MPH (Faculty); John M. Popovich Jr., PhD, DPT, ATC (Faculty) 25
41. **MSU and EMU College Student Caregiver Study.** Nicholas Hallenbeck (Medical Student); Sowmyaw Nakkina (Medical Student); Clare Luz, PhD (Faculty) 25
42. **Personalized Research Advisor/Student Recommendation in Medical Education Using Large Language Models.** Na Dai (Medical Student); Doha Amin; Reshma Dukkipati; Rula Ibrahim; Shweta Bhavsar; Janice Schwartz (Faculty); Carolina Restini (Faculty) 26
43. **Developing Communication Skills Through Community-Engaged Teaching and Learning Services is Aligned With Humanized Cognitive Factors Impacting OMS Self-Perception.** Selena Tiberio (Medical Student); Carolina Restini, PharmD, PhD; Carrie Nazaroff, PhD; Gabriella Carr (Medical Student); David Ibrahim (Medical Student); Sami Abdelaziz (Medical Student); Ryan Matusik, (Medical Student); Elise Torres (Medical Student) 27
44. **The Impact of Prior Authorization Removal on Opioid Use Disorder Treatment in Michigan.** Nirmeen Chouaib, BS (Medical Student); Marina Haque MD, MPH (Resident); Wael Saasouh, MD (Faculty); Noura Chbeir, MA (Policy Consultant) 27
45. **Cardiac Relaxation Effects Caused by the Myosin-Specific Drug Omecamtiv Mercarbil and Myosin Isoform Content.** Benjamin M Cavanaugh (Medical Student); Alexandra R Matus, Melissa J Bukowski, Brianna M Schick, Po-Jen Chiang, Donal O’Leary, Joseph T Mannozzi, Charles S Chung, PhD (Faculty) 28

Below are digital abstracts of the oral and poster presentations at the MSUCOM First Annual Research Day:

## 1

## DIFFERENTIAL EFFECT OF AGE ON MORTALITY IN AFRICAN AMERICANS INFECTED WITH THE SARS-COV-2 VIRUS IN THE UNITED STATES

**Kevin Kwamin, MD (Resident);** Dylan Arroyo; Blake Hardin, MS-I; Hope Ring, MD; Daniel Keyes; MD, MPH (Faculty).

SCS-affiliated Resident Physician or Fellow; Trinity Health-Livonia, MI.

### BACKGROUND

It has been previously reported that African Americans (AA), had a disproportionately higher rate of infection and mortality during the COVID-19 pandemic. This study ex- amines the effect of age on mortality disparities.

### OBJECTIVES

To determine if there is a difference in mortality rates be- tween the different age groups of African Americans and White people.

### METHODS

This was a retrospective cohort design. A multistate model of large hospital system electronic health records (EHRs) included patients during the pandemic (January 1, 2019, to March 31, 2022), of unique first-time test-positive patients ages 10-90 years. The primary outcome variable was mortality as a function of age. Logistic regression models were based on predictive factors of age, sex, race, week of admission, Charlson Comorbidity Index (CCI), and BMI. When the log odds are positive, there is a disadvantage for AAs.

### RESULTS

Starting with 1,520,483 records, the final dataset for covid positive subjects had 330,648 encounters for 171,125 dis- tinct people. There were 7,155 deaths, with a crude death rate of 4.2%. Following a positive COVID-19 test result, the mortality disparity almost always either disfavored AAs or showed no statistical evidence of a disparity. The disparity tended to increase with age and decrease as the pandemic progressed. The greatest disadvantage for AAs is for older patients near the end of 2020, where the log odds ratio is around 0.6, corresponding to 1.8-fold greater odds of death for AAs. Statistically elevated mortality in AAs was evident for people over 55 from the beginning of the pandemic until mid-May of 2020.

### CONCLUSION

The most significant racial disparity noted during the pandemic is increased mortality for older African Americans during the early pandemic. It is essential to investigate the reasons for older African Americans exhibiting greater vulnerabilities to provide effective preventive strategies for these risk differences.

Email of Presenting Author: Kevin.Kwamin@trinity-health.org

## 2

## DEVELOPMENT AND EVALUATION OF A VIRTUAL REALITY SIMULATION ENVIRONMENT FOR CARDIOLOGY EDUCATION: A PILOT STUDY

**Chris Jacob, DO (Fellow)**; Brilio (Jon) Mojares, MD, FACC (Faculty).

SCS-affiliated Resident Physician or Fellow; Ascension Macomb-Oakland Hospital, MI.

### BACKGROUND

Anatomic education classically depends on resources like cadaveric specimens and textbook atlases. In interventional cardiology, 3D coronary anatomy must be interpreted via 2D angiographic images that change based on the positioning of the C-arm, and optimal positioning is required to view the relevant parts of the anatomy. Traditionally, this has been learned by assisting in live cases. However, this knowledge acquisition is especially challenging for trainees given the inherent variation between patients, the stress of invasive high-risk procedures, and the radiation exposure hazard linked to every angiography image. We sought to evaluate the feasibility and effectiveness of a virtual reality (VR) based simulation for catheterization education.

### METHODS

We programmed a VR simulation program using the Oculus Quest 2. The simulation included a VR catheterization lab including patient, C-arm and angiography screen. The user is able to look around the simulation in lifelike motion and control the C-arm which updates the angiography in real- time. Participants performed a pretest, then used the simulation for 15 to 30 minutes, and then completed a posttest. The tests assessed knowledge, comfort level and ease of use. The study was IRB approved.

### RESULTS

Fifteen internal medicine residents and first year cardiology fellows were recruited because they had minimal to no catheterization lab exposure. Participants averaged 17 minutes in the simulation. The mean pretest score was 9% and rose to 49% posttest. By paired sample T test, this was statistically significant at p<0.0001. The mean comfort score for interpreting angiography was 2 out of 7 at baseline, which rose to 4 out of 7 after intervention. Ease of using the simulation was rated at 6 out of 7.

### CONCLUSIONS

We have shown that the VR simulation program we de- signed shows promise as an educational tool for catheterization training. This method allows for stress free training without the potential of harming a real patient or unnecessary radiation exposure. Further improvement of the soft- ware is planned to further enhance the realism and possibly add the option of virtually performing interventional procedures. Future research can also be performed on larger groups of trainees or at different training sites.

Email of Presenting Author: chris.jacob@ascension.org

## 3

## BIOCHEMICAL, STRUCTURAL, AND ALLERGENIC CHARACTERIZATION OF PLANT AND MITE PROFILINS

**Andrea O’Malley, PhD (Post-doctoral Researcher)**; Avery Carriuolo, BS; Sahana Sankaran, BS; Krzysztof Kowal, MD; Maksymilian Chruszcz, PhD (Faculty).

Trainee with MSUCOM Faculty; MSUCOM.

### INTRODUCTION

Profilins are small (13-15 kDa) proteins whose primary function is the regulation of cytoskeletal dynamics, binding actin, proline-rich peptides, and phosphoinositides for this purpose. Profilins have a conserved structure, with three alpha helices and seven beta sheets forming a single do- main. Profilins are found ubiquitously in eukaryotes, with the most commonly studied profilins found in humans and plants. Mutants of human profilin, for example, have been found to be relevant in diseases such as amyotrophic lateral sclerosis. However, profilins are most studied in the context of their status as plant allergens.

### METHODS

Here, we focus on the biochemical and structural characterization of several profilin allergens using differential scanning fluorimetry, size-exclusion chromatography, dynamic light scattering, and x-ray crystallography. Additionally, we evaluate the allergenicity of two profilins from mites with ELISA and inhibition ELISA.

### RESULTS & CONCLUSIONS

Using cysteine-serine mutants, we show that conserved cysteine residues are relevant in the stability and oligomerization of plant and mite profilins. We were also able to determine new structures of several profilins from plants, including those of profilins from Timothy grass, apple, and melon. Additionally, we present the first structures of mite profilins from Arachnida. Finally, the initial evaluation of the allergenicity of two mite profilins demonstrate a lack of cross-reactivity between plant and mite profilins. Further studies on large groups of patients should allow for an evaluation of possible clinical relevance of mite profilins in mite-allergic patients.

Email of Presenting Author: omalle89@msu.edu

## 4

## SKIN INJURY PROGRESSION FOLLOWING DERMAL NITROGEN MUSTARD EXPOSURE IN C57BL/6 AND MAST CELL DEFICIENT MICE

**Andrew Roney (Trainee with Faculty)^1^**, Dinesh Goswami^1^, Ebenezar Okoyeocha^1^, Steve Lundback^1^, Holly Wright^1^, Omid Madadgar^1^, Ellen Kim^1^, Jared M Brown^2^, Neera Tewari-Singh.^1^

^1^MSUCOM- Department of Pharmacology and Toxicology

^2^Department of Pharmaceutical Sciences, University of Colorado Denver, Aurora, CO.

Trainee with MSUCOM Faculty; MSUCOM.

Cutaneous vesicant exposure results in severe skin injuries including blistering, which can in turn result in secondary infections, and lead to systemic toxicity at high doses. Sul- fur mustard (SM) has been the most widely used chemical threat agent since World War I and remains a potential chemical threat. However, we lack effective therapies for SM induced skin and hematologic toxicity leading to systemic and long-term illnesses in exposed victims, especially from the Gulf War. Mast cells, innate immune cells with a regulatory role in immunity and acute inflammatory responses, have been implicated in mustard-induced injury in previous studies. However, their role has not been elucidated in mustard vesicant-induced acute and long-term effects in skin and systemic toxicity. Characterizing the role of mast cells in mustard toxicity could aid the development of effective therapies for SM exposure. In this study we aimed to investigate the role of mast cells by com- paring mustard vesicant-induced skin injury in wild-type (WT; C57BL/6) and mast cell-deficient (MCD; B6.Cg-KitW- sh/HNihrJaeBsmJ) mice.

Male WT and MCD mice were exposed topically to 0.5 mg of nitrogen mustard (NM, a structural analog of SM) in 100 mL of acetone. Survival was monitored and clinical assessments (body weight, skin bifold thickness, erythema, edema, and necrosis) were carried out at various timepoints post-exposure and images were taken.

NM exposure caused cutaneous injury, inflammation, and increased mast cell degranulation in WT mice com- pared to controls. The post-exposure survival rate was significantly greater for MCD mice than WT mice, with 50% and 33% survival at 14 days post-exposure, respectively. Body weight loss, edema and erythema were less severe in MCD mice compared to WT mice.

Overall, NM exposure resulted in less severe skin injury, lower mortality, delayed weight loss and in lower severity of other assessed clinical parameters in MCD mice compared with WT mice. Overall, these results indicate that mast cells play a role in mustard-induced skin injury and long-term toxic effects; histological and molecular analyses are being carried out to decipher if mast cells are beneficial targets for therapeutic interventions to ameliorate toxicity from mustard vesicant exposures.

Email of Presenting Author: roneyand@msu.edu

## 5

## MIMICKING GENDER-AFFIRMING TREATMENT OF PUBERTAL SUPPRESSION FOLLOWED BY TESTOSTERONE ON BONE GROWTH IN FEMALE-BORN MICE

**Brandon W. Henry (Medical Student)**; Cynthia Dela Cruz, PhD (Post-doc Fellow); Robert W. Goulet, PhD; Bonnie T. Nolan; Vasantha Padmanabhan, PhD; Molly Moravek, MD; Ariella Shikanov, PhD, FACP; Megan L. Killian, PhD, FACP.

MSUCOM Medical Student; MSUCOM.

### BACKGROUND

Sex steroids are crucial in attainment and maintenance of peak bone density. Inhibition of the hypo- thamaopituitary gondal axis can lead to a hypoestrogenic state, which may influence bone homeostasis. In this study, we induced a hypoestrogenic state using gonadotropin re- lease hormone agonist (GnRHa) to examine effects on femoral bone quality in female-born mice subjected to pubertal suppression, followed by testosterone (T) treatment. Methods: 22 C57BL/6N female mice (age: postnatal day 26) were assigned to four experimental groups: Sham surgery (controls; n=7), GnRHa-only treatment (n=3), post-T treatment (n=7), and GnRHa + post-T treatment (n=5). For the GnRHa and GnRHa + post-T groups, mice were implanted with GnRHa. Control and post-T groups under- went a sham surgery. After 3-weeks of GnRHA treatment, mice were implanted with T or empty silastic tubing. After 6-weeks of T treatment, mice were explanted, euthanized, and imaged using nano-computed tomography to assess bone morphology.

### RESULTS

Experimental group treatment, during peri-pubertal growth, did not influence mouse weight at experimental endpoints. Femur length was significantly shorter in post-T treated compared to GnRHa-only mice. Trabecular bone volume fraction (BV/TV) was higher for post-T treated groups compared to non-T-treated groups. Additionally, BV, was significantly higher compared to control for the T and GnRHa + T groups. Also, BV of the GnRHa- only group was significantly smaller than T and GnRHa + post-T treatment groups. Additionally, we found increased trabecular number in T-treated mice compared to Control and GnRHa-only treated mice.

### CONCLUSIONS

GnRHa-treatment with post-T treatment during peripubertal growth had a strong effect on trabecular bone but only a marginal effect on cortical bone. We found that T administration led to shorter bone length, which could be explained by androgen-mediated delay in closure of growth plate, potentiating continued growth at later stages of maturation. Typical therapy in transmasculine youth consist of puberty suppressors and androgen treatment. It is important to understand if and how pubertal suppression influences skeletal development to help guide clinical treatment for transgender patients.

Email of Presenting Author: henrybr3@msu.edu

## 6

## STRUCTURAL CHARACTERIZATION OF COMPLEX BETWEEN HOUSE DUST MITE ALLERGEN DER P2 AND HUMAN-DERIVED IGE MAB 4C8 FAB

**Kriti Khatri, PhD (Professional Aide)^1,2^**; Alyssa Ball, PhD^3^; Jill Glesner^3^; Anna Pomés, PhD^3^; Scott Alan Smith, PhD, MD^4^; Maksymilian Chruszcz, PhD (Faculty)^1^.

^1^Michigan State University, East Lansing, MI 48824, USA

^2^University of South Carolina, Columbia, SC 29208, USA

^3^InBio, Charlottesville, VA 22903, USA

^4^Vanderbilt University Medical Center, Nashville, TN 37235, USA

Trainee with MSUCOM Faculty; Biochemistry and Molecular Biology.

### BACKGROUND

Dust mite allergy affects 10-30% of the world’s population. Der p 2 from Dermatophagoides pteronyssinus is a major allergen that causes allergic conditions in more than 80% of house dust mite-allergic individuals. 3D structures representing Der p 2-IgEs interactions allow for Der p 2 epitope mapping. Information on IgE epitopes is essential for designing mutant allergens, which can be recommended for allergy immunotherapy.

### METHODS

Human IgE (hIgE) mAb 4C8 was isolated from hybridomas obtained from a clinically diagnosed house dust mite allergic patient. 4C8 Fab with the correctly paired heavy and light chains was sequenced and expressed in CHO cells. Der p 2 and mutants were expressed in Pichia pastoris. Purified 4C8 Fab and Der p 2 were mixed in a 1:1 molar ratio for crystallization. X-ray crystallography was employed to elucidate the structure of Der p 2 bound to 4C8 Fab. Immunoassays were used to assess the binding inter- action of mutants towards the antibody.

### RESULTS

The crystal structure of Der p 2 in complex with hIgE mAb 4C8 Fab was elucidated at a resolution of 3.0 Å. The structure reveals monomeric Der p 2 interacting with two 4C8 Fabs. The 4C8 epitope had not been previously identified and does not overlap with known Der p 2 binding sites of hIgE 2F10 and murine IgG 7A1.

### CONCLUSIONS

The structure of the Der p 2-4C8 Fab com- plex reveals a previously unidentified epitope on Der p 2. The identification of this IgE epitope allows for the rational design of hypoallergens with reduced IgE affinity.

Email of Presenting Author: khatrikr@msu.edu

## 7

## POLYUNSATURATED FATTY ACID METABOLISM INVOLVEMENT IN AGE-ASSOCIATED NEURODEGENERATION

**Jennifer Hinman (PhD Student)**; Morteza Sarparast (Graduate Student); Elham Pourmand (Graduate Student); Jamie Alan, RPh, PharmD, PhD (Faculty); Kin Sing Stephen Lee, PhD (Faculty).

Trainee with MSUCOM Faculty; MSU Department of Chemistry.

Common neurodegenerative diseases (ND) such as Alzheimer’s (AD) and Parkinson’s (PD) are estimated to currently affect 6.5 million and nearly one million people, respectively, within the United States alone. However, the mechanism behind AD and PD pathogenesis remains un- known and current treatment for these diseases are only palliative. Recent evidence has indicated that aging in- creases the risk of cognitive decline and exacerbate neurological symptoms leading to neurodegenerative diseases. Recent studies found that epoxide hydrolases in the oxidative ω-3 and ω-6 polyunsaturated fatty acid (PUFA) metabolic pathways and their enzymatic products are upregulated in AD patients. Therefore, we hypothesize that aging affects age-associated neurodegeneration by modulating specific cytochrome P450-epoxide hydrolase (CYP-EH) metabolism.

To investigate how PUFA metabolism is modulated by aging that then affects age-associated neurodegeneration in human or mammal studies would be difficult due to time and cost of colleting these samples; therefore, we propose to use model organism Caenorhabditis elegans. C. elegans are beneficial due to their short lifespan, low cost and maintenance, transparent body allows for live imaging, and genetically malleable. As well, C. elegans have con- served neuron signaling and PUDA oxidative metabolism and ~65% homology for human disease genes.

To investigate these effects, fluorescent neuronal imaging will be assessed on transgenic strains in which either the cholinergic (Punc-17::gfp) or GABAergic (Punc-25::gfp) neurons are tagged by green fluorescent protein to identify physical signs of ND over aging while the oxylipin profile will be collected from a batch of age-synchronized wild- type worms and analyzed via HPLC-MS/MS to identify global PUFA metabolite changes. We have currently demonstrated a trend of increasing prevalence of neurodegeneration throughout aging in both transgenic strains as well as notable changes to the epoxy/dihydroxy ratio in middle aged worms and significant changes in ω-3 dihydroxy metabolites with aging. Further studies may track changes with supplementation of these compounds while other studies will be performed to determine the cell death mechanisms involved in age-associated neurodegeneration. Overall, assessing the crosstalk between aging and PUFA metabolism in C. elegans will begin to increase our understanding of the pathogenesis behind neurodegenerative diseases such as AD and PD.

Email of Presenting Author: hinmanje@msu.edu

## 8

## UNRAVELLING THE SPECIFICITY OF BMP 9 AND BMP 10 SIGNALING VIA BMPR2 IN SMAD 1/5/8 ACTIVATION

**Kit Yee Chu (DO/PhD Student)**; Dr Erik Martinez- Hackert (Faculty, PI).

MSUCOM Medical Student; MSUCOM.

### BACKGROUND

BMP9 and BMP10 are members of the transforming growth factor-beta (TGF-β) superfamily of cytokines. These growth factors (GFs) play a crucial role in the development and maintenance of the pulmonary vasculature. Their dysregulation has been implicated in the pathogenesis of pulmonary arterial hypertension (PAH). In general, TGF-β GFs bind and form signaling complexes with TGF-β type II and type I receptors, leading to the activation of either SMAD 1/5/8 or SMAD 2/3 signaling pathways.

Many studies have shown that BMP9 and BMP10 can bind to type II receptors, including BMPR2, ActRIIA, and ActRIIB, and activate SMAD 1/5/8 signaling. However, it is unknown whether they signal through all receptors or form only one functional signaling complex. This study aims to demonstrate whether BMP9 and BMP10 have any specificity toward the type II receptors for SMAD 1/5/8 activation.

### METHODS

We studied the receptor-GF interaction using surface plasmon resonance. We measured the activation of the SMAD 1/5/8 and SMAD 2/3 signaling pathways using immunoblotting in pulmonary arterial endothelial cells (PAECs) and determined the effect of BMP9 and BMP10 on pulmonary cell proliferation using BrdU cell proliferation assays. We also use reporter gene assays in stably transfected HEK 293 cells to measure the two signaling pathways.

### RESULTS

We found that BMP9 and BMP10 increase pro- liferation in PAECs. Also, BMP9 and BMP10 can bind to all three type II receptors, BMPR2, ActRIIA, and ActRIIB. When we treated the PAECs with BMP9 and BMP10 with or without Bimagrumab (BiMAB), which is an antibody that blocks the ectodomain of ActRIIA and ActRIIB, we found that in the presence of BiMAB, there is no inhibition of SMAD 1/5/8 signaling induced by BMP9 and BMP10. In addition, BiMAB does not inhibit BMP9 and BMP10 induced cell proliferation.

### CONCLUSION

The study demonstrated that although BMP9 and BMP10 can bind to all three type II receptors, they signal mostly via BMPR2 to activate the SMAD 1/5/8 pathway.

Email of Presenting Author: chukit@msu.edu

## 9

## WITHOUT A TRACE: THE PIRIN AND NF-KB INTERACTION

**Melissa Meschkewitz, BS (DO/PhD Student)**; Erika M Lisabeth, PhD (Faculty); Richard R Neubig, MD, PhD (Faculty).

MSUCOM Medical Student; MSUCOM.

Pirin is a non-heme iron binding protein and was recently identified as a protein target of the CCG-20391 series of small molecules. The CCG compounds were originally discovered in a high-throughput Rho/MRTF/SRF dependent luciferase screen and have since been shown to have anti- fibrotic and anti-metastatic properties, but the mechanism of how Pirin affects MRTF/SRF signaling has yet to be identified. Pirin has been shown to have a role in many different cellular processes, namely epithelial to mesenchymal transition, ferroptosis, senescence, and more. Interestingly, Pirin has also been thought to be a nuclear co-transcription factor to various transcription factors, one of which is the NF-κB family of proteins. Therefore, our hypothesis was that modulation of Pirin through the binding of the CCG compounds may modulate NF-κB pro-inflammatory signaling. To investigate this, we used recombinant biochemical techniques like Fluorescence Polarization, Size Exclusion Chromatography, and Gel Shift Assays to investigate the interaction between Pirin and NF-κB. Additionally, we developed a novel Pirin knockout (KO) mouse model to further determine the functional relationship between Pirin and NF-κB. Surprisingly, we are not able to detect a stable complex of Pirin and the RelA subunit of NF-κB nor do we observe any modulation of inflammatory genes by qPCR in a Pirin KO mouse model. Upon closer investigation of the cellular location of Pirin we found that Pirin is localized in the cytoplasm and does not translocate to the nucleus upon NF-κB activation. We therefore conclude that, based on our observations, Pirin has cellular functions other than a co-transcription factor. These functions may include modulation of MRTF/SRF signaling and actin polymerization, but do not affect NF-κB signaling.

This work is supported by NIH RO1 GM115459-07

Email of Presenting Author: meschkew@msu.edu

## 10

## DEVELOPMENT OF NOVEL IMMUNOTHERAPY USING A VIRAL MULTI-GENE DELIVERY SYSTEM TO TREAT HUMAN PAPILLOMAVIRUS-POSITIVE HEAD AND NECK CANCER

**Nicholas S. Giacobbi, MS (DO/PhD Student)**; Harrison Nabors (Undergraduate Student); Shreya Mullapudi, BS (Medical Student); Dohun Pyeon, PhD (Faculty).

MSUCOM Medical Student; MSUCOM.

### BACKGROUND

Since the 1990s, there has been an epi- demic increase in the incidence of human papillomavirus- positive head and neck squamous cell carcinoma (HPV+ HNSCC). Disease management is challenged by high rates of treatment-related morbidity and at least 20% of patients failing to respond to current treatment modalities. Thus, the development of new treatments for HPV+ HNSCC is necessary.

We have observed that the chemokine, CXCL14, is highly downregulated in HPV+ cancers. Interestingly, restoration of CXCL14 enhances antigen presentation by increasing MHC-I and T-cell infiltration into tumors, resulting in tumor suppression in vivo. Because therapy refractory HPV+ HNSCC cases demonstrate limited antigen presentation and T-cell infiltration into tumors, we hypothesize that CXCL14 (re)introduction could be used as a novel immunotherapeutic.

### METHODS

I have cloned a therapeutic transgene cassette to deliver CXCL14 and HPV E6/E7 epitopes to tumors via an adenoviral vector (Ad5). In addition, I have also evaluated tumor-specific promoters to enhance cancer cell specific transgene expression, relative to normal cells, using a luciferase reporter system. With the completion of adenoviral production, the full cassette combining CXCL14 and E6/ E7 epitopes, CXCL14 alone, or E6/E7 epitopes alone, will be evaluated for their effect on in vivo tumor growth and T cell infiltration by intratumoral injection into tumor-bearing immunocompetent syngeneic mice.

### RESULTS

Expression of both CXCL14 and E6/E7 epitopes were successfully detected from transient transfection in 293FT cells. Infection by Ad5-CXCL14 in HPV+ HNSCC cells showed CXCL14 expression 24 hours post infection. I have also successfully mapped minimal sites necessary for the HPV+ HNSCC specific activation of the mouse and human CDKN2A (p16) promoter. The identified minimal promoter unit will be utilized as a synthetic promoter for tumor specific expression of the therapeutic cassette in vivo.

### CONCLUSIONS

Delivery of CXCL14 and E6/E7 epitopes un- der the control of a synthetic tumor-specific promoter is a promising strategy for novel adenoviral transgene immunotherapy to treat HPV+ HNSCC.

Email of Presenting Author: giacobbi@msu.edu

## 11

## BONE BENEFITS OF DIETARY PRUNE IN HEALTHY FEMALE MICE ARE TIME DEPENDENT AND ASSOCIATED WITH DRASTIC SHIFTS IN GUT MICROBIOTA COMPOSITION

**Nicholas J. Chargo (DO/PhD Student)**; Kavisha Patel; Leon Regnery; Robert A. Quinn, PhD; Narayanan Parameswaran, PhD (Faculty); Laura R. McCabe, PhD (Faculty).

MSUCOM Medical Student; MSUCOM.

### INTRODUCTION

The gut microbiota plays a critical role in bone health. Prunes (dried plums), a prebiotic and nutraceutical, alter the gut microbiota, prevent and reverse primary osteoporosis in rodents and prevent further bone loss in post- menopausal women. However, the temporal relationship and microbiota-related mechanisms by which these bone benefits occur are poorly understood.

### METHODS

To better understand the relationship between prune-induced gut microbiota changes and bone health, we treated healthy 16-week-old C57BL/6J female mice with control (AIN-93M) or 25% prune (w/w) diets for up to 8 weeks. Mice were humanely euthanized at various timepoints after designated periods on the diet or following a period of discontinuation and general body parameters, bones, and colonic feces were collected. Trabecular bone from the distal femur and L4 vertebral body were analyzed via microcomputed tomography (uCT). Colonic fecal samples were processed for sequencing of the 16S rRNA gene (V4 region) with subsequent analysis in Qiita.

### RESULTS

Results show that 25% prune diet significantly increases distal femur trabecular bone volume/total volume% (BV/ TV%) in a time dependent manner with increases of ~50% at 10 days (p<0.0001), ~250% at 4 weeks (p<0.0001), and~300% at 8 weeks (p<0.0001). The gut microbiota com- position was also significantly altered at each time point (p=0.001) compared to controls as assessed by weighted UniFrac distances and PERMANOVA testing. Interestingly, when mice were treated with the 25% prune diet for 10 days and then switched to the control diet for 20 additional days, the bone benefits were completely lost (p=0.9842) and the gut microbiota composition returned to normal (p=0.338). No differences in cortical bone were observed at any time- point. Further analyses are underway to determine specific changes in microbiota composition.

### CONCLUSION

This study demonstrates the time-dependent nature of prune-induced bone benefits and the associated shifts in gut microbiota composition. We also demonstrate that dis- continuation of the prune diet causes loss of prune-induced bone benefits, and the gut microbiota shifts back to the control composition. Further studies are needed to better understand if gut microbiota changes are driving the prune-induced bone benefits.

Email of Presenting Author: chargoni@msu.edu

## 12

## PROGRESSION OF CHLOROPICRIN-INDUCED OCULAR INJURY: CLINICAL AND BIOLOGICAL EFFECTS

**Okoyeocha OM Ebenezar (PhD Student)**; Andrew Roney; Dinesh G Goswami; Lauren Gizinski; Zubeida Abouarko; J Mark Petrash; Dodd Sledge; András M Komáromy; Karen Liby; Neera Tewari-Singh (Faculty).

Trainee with MSUCOM Faculty; MSUCOM.

Chloropicrin (CP) vapor exposure via warfare, occupational, or accidental routes results in severe ocular injury, especially to the cornea. Employed during World War I and currently used as a pesticide and fumigating agent, CP re- mains a potential chemical threat agent. However, studies on ocular injury progression and underlying mechanisms in a relevant in vivo model are lacking. This study shows the clinical and biological progression of CP-induced ocular in- jury while testing different CP concentrations in mice to develop a relevant injury model. Using an ocular only vapor exposure mouse model, the left eye of male BALB/c mice were exposed to 20% CP for 1 min or 30 sec (0.4 mg of CP) or 10% CP (0.2 mg of CP) and the right eye served as a control. Mice were euthanized at day 25 post exposure and the eyes were harvested to further study the corneal in- jury. CP caused a significant increase in corneal ulceration and eyelid swelling which resolved by day 14 post exposure. However, CP caused an unresolved significant increase in the corneal opacity and neovascularization. Development of corneal hydrops (bullae due to corneal edema) and hyphemia (blood accumulation in the anterior chamber) were observed as advanced CP effects. Histopathological analyses showed a significant CP-induced decrease in corneal epithelial thickness, and an increase in stromal thickness with more pronounced injury including stromal fibrosis, edema, neovascularization, anterior and posterior synechia and in- filtration of immune cells. Loss of the corneal endothelial cells could be associated with the CP-induced corneal edema with fluid buildup causing hydrops that can con- tribute to cloudy vision, corneal scarring and eye pain. Damage to the uveal vessels from CP might be further re- sponsible for corneal opacity and hyphemia. Although exposure to 20% CP for 1 min caused more eyelid edema, ulceration and hyphemia, other effects were similar in all CP exposures. These novel findings following CP ocular expo- sure in a mouse model will be useful in further ocular in- jury model development and in pathophysiological studies to identify molecular targets for therapeutic interventions.

Email of Presenting Author: okoyeoch@msu.edu

## 13

## THE ROLE OF LPA IN GLIAL SIGNALING OF VISCERAL PAIN

**Visha Parmar (DO/PhD Student)**; Brian Gulbransen, Ph.D. (Faculty, Principal Investigator).

MSUCOM Medical Student; MSUCOM Department of Physiology.

Visceral pain is the most common gastrointestinal issue and a hallmark clinical feature of inflammatory bowel dis- ease and irritable bowel syndrome. Visceral hypersensitivity is a major mechanism that contributes to pain but the processes leading to visceral hypersensitivity are not understood. Enteric glia surround sensory nerve terminals in the intestine and exhibit bi-directional communication with nociceptors. Our recent discoveries show that enteric glia are highly responsive to the bioactive lipid lysophosphatidic acid (LPA), which is known to contribute to neuropathic pain. Therefore, we hypothesize that LPA con- tributes to visceral hypersensitivity through glial mechanisms that sensitize Trpv1+ nociceptors. To deter- mine if LPA activates or sensitizes Trpv1+ neurons, in vitro Ca2+ imaging was conducted in whole mounts of colonic tissue from Trpv1Cre; GCaMP5g-tdT mice that express the genetically encoded Ca2+ sensor GCaMP5 in Trpv1+ sensory neurons (nociceptors). GCaMP5 fluorescence (F) in Trpv1+ nerve terminals innervating the myenteric plexus of distal colon was measured at baseline, following expo- sure to the Trpv1 agonist capsaicin, following a LPA challenge, and in response to capsaicin following exposure to LPA. ΔF/F0 was quantified based on number of frames and maximum response peaks were analyzed. Sox10CreERT2; Lpar1f/f mice were developed and used to induce a conditional deletion of LPAR1 in glia following a tamoxifen diet for two weeks to evaluate how survivor- ship, body weight, and colon length may vary between wild type and LPAR1 KO mice. Preliminary results suggest that LPA alone is not sufficient to activate Ca2+ responses in Trpv1+ neurons. However, capsaicin responses following exposure to LPA tended to be larger than those to capsaicin in naïve samples, although this effect has not reached statistical significance due to the underpowered nature of pre- liminary experiments. Deleting glial Lpra1 in Sox10CreERT2; Lpar1f/f mice did not have an appreciable effect on survivorship or overt pathophysiology. Based on these preliminary results, LPA is a candidate mediator of glial-driven visceral hypersensitivity, but future studies are needed to understand how glial LPAR1 signaling con- tributes to gut health and disease.

Email of Presenting Author: parmarv2@msu.edu

## 14

## MASSAGE-LIKE STROKING PRODUCES ANALGESIA IN MICE

**Zachary M.S. Waarala (Medical Student)**; Logan Comins (Medical Student); Geoffrey Laumet, PhD (Faculty).

MSUCOM Medical Student; MSUCOM.

### BACKGROUND

Massage-like stroking (MLS) induces pain relief in humans. MLS is similar to long-used manual medicine treatments such as massage, acupuncture, MFS, and several osteopathic manipulative treatments such as myofascial release and soft tissue technique. MLS activates the endogenous analgesic system without side-effects making it an optimal therapy in front of the current pain crisis and opioid epi- demic. MLS is not convenient for long-term treatment, but the lack of understanding of the underlying molecular and cellular mechanisms activated by MSL prevents leverage of the endogenous analgesic system for chronic pain treatment. To decipher these mechanisms, it is necessary to establish a rodent model of MLS.

### METHODS

The MLS procedure had mice receiving 20 strokes per five- minute intervals over 60-minutes at a rate of three centimeters/second in a cephalo-caudal direction. Pain response was measured both pre- and post-treatment via hot plate testing (latency to react to heat stimuli). Mice were separated into groups of massage, hold-only, or control. Elevated Plus Maze (EPM) was conducted to evaluate potential stress. To separate physiologic mechanisms from psychological ones, mice underwent anesthetization with isoflurane, where one group received MLS while the other received only anesthesia.

### RESULTS

Mice that were subjected to the MLS protocol have an in- creased latency to heat stimuli (p<0.0001) compared to control, which indicates an analgesic effect of MLS. These effects were observed in both males (p<0.01) and females (p<0.0001). The analgesic effect of MLS is conserved when MLS procedure and post-treatment hot plate testing done by different experimenters(p<0.001) compared to control. Hold-only mice, those which do not receive the MLS, do not show analgesic effects. MLS is not associated with behavioral signs of stress as measured by EPM. Performing MLS under isoflurane anesthesia is still inducing analgesia (p<0.05), suggesting that MLS-induced analgesia is sup- ported by physiological rather than psychological factors.

### CONCLUSION

This study established a novel preclinical model of MLS-induced analgesia in wild type mice. This model will allow us to decipher the cellular and molecular mechanisms of MLS.

Email of Presenting Author: waaralaz@msu.edu

## 15

## A COMPLICATED CASE OF HEPARIN-INDUCED THROMBOCYTOPENIA

**Ananda Vigneswari Anebarassou, MD, MPH (Resident)**; Jeevana Sreeja Ambati MD (Resident); Sujatha Kambhatla MD (Faculty).

SCS-affiliated Resident Physician or Fellow; Garden City Hospital, MI.

### BACKGROUND

Heparin Induced Thrombocytopenia with thrombosis (HITT) is a well-known complication occurring in patients exposed to unfractionated or LMW heparin. Most of these patients need to be on non-heparin anticoagulants for at least 3 - 6 months. Limb threatening complications occur infrequently leading to requirement of emergency endovascular intervention.

### CASE DESCRIPTION

A 74-year-old morbidly obese male with past medical history of atrial fibrillation (on warfarin), OSA, gout, chronic venous insufficiency, was admitted to the hospital from inpatient rehabilitation center with declining tolerance to physical therapy and acute hypoxic respiratory failure. He was in the rehabilitation center for continued therapy following lumbar laminectomy, indicated for chronic debilitating de- generative disc disease. His long-term anticoagulation was briefly interrupted during the perioperative period. On POD 2, the patient was restarted on warfarin, in addition to heparin, as the INR was below therapeutic range. 8 days after initiation of heparin, the patient was noted to have severe thrombocytopenia (platelet count <10K) with no evidence of mucocutaneous bleeding. Simultaneously, the patient was found to have superficial venous thromboses of the left upper extremity, acute massive DVT of bilateral lower extremities and a sub massive, high-risk PE. He was considered to be at high probability for HIT. All heparin products were withdrawn and substituted with therapeutic dose of fondaparinux. HIT antibody panel was sent. Evaluation of the lower limb arterial system did not show any hemodynamically significant stenosis. The HIT antibody panel was positive with a titer of 2.09 OD. The patient was started on argatroban infusion. After multiple attempts, he was transferred to a higher center capable of vascular surgical intervention using non heparin products where he underwent mechanical thrombectomy of the left lower extremity. At the conclusion of the procedure, the patient coded. ROSC was never achieved despite early administration of tPA, and the patient was pronounced dead after 40 mins of total resuscitation.

### CONCLUSION

Widespread adoption of endo-vascular techniques using non heparin products can avoid delays in care for patients with complicated HITT. More studies involving medically complex patients are needed to validate the risks and benefits of timely endovascular intervention in HITT.

Email of Presenting Author: aanebarassou@phsi.us

## 16

## A LETHAL CASE OF ATYPICAL CHEST PAIN

**Andrew Victor, MD (Resident)**; Hadeel Barrawi, DO (Res- ident); Dylan Mooney, DO (Faculty).

SCS-affiliated Resident Physician or Fellow; Family Medicine, Ascension Genesys, MI.

### CASE DESCRIPTION

A 48-year-old African American female with a complaint of shortness of breath and retrosternal chest pain presented to the Emergency Department (ED). She recently under- went a catheter ablation for paroxysmal atrial fibrillation (AF) one month prior. Following her ablation, the patient had multiple visits to the ED as well as outpatient follow up appointments. Throughout this time the patient had been complaining of sustained and unrelenting symptoms that included chest pain, odynophagia, nausea and vomiting. A series of investigations that included a transthoracic echo, CTA of the chest to rule out pulmonary embolism, ECGs and various laboratory tests were overall normal. Eventually the patient did present a final time to the ED. This time however, she was septic with bacteremia and fungemia as well as requiring intubation for respiratory failure. She was also found to have a left atrial mass. When the decision was made to transfer her to a tertiary care center, she was pronounced brain dead on arrival. It was later determined that the patient had a rare but known complication of catheter ablation known as an atrial-esophageal fistula. It is estimated to occur in <0.1 - 0.25% of cases. It can occur anytime up to 6 weeks after the procedure. Most of the signs and symptoms are relatively non-specific. It is paramount to avoid an upper endoscopy for evaluation as insufflation may generate an air embolism. Mortality from this complication is 100% if left untreated.

### CONCLUSION

This case highlights a lethal and rare complication from a routine procedure for a relatively common condition of AF. With the symptoms being non-specific and despite the patient being evaluated by multiple healthcare providers in various settings, the diagnosis was made too late. This can be due to many factors associated with the healthcare environment, communication, access to resources, assessment by providers as well as inherent and unknowing bias and judgment based on a patient’s race and/or socioeconomic status. Earlier diagnosis of this condition may have prevented this morbid outcome and as such, mortality from this treatment complication, remains 100%.

Email of Presenting Author: andrew.victor@ascension.org

## 17

## SPONTANEOUS BACTERIAL PERITONITIS AND PNEUMATOSIS COLI IN THE PRESENCE OF PASTEURELLA MULTOCIDA INFECTION WITHOUT EVIDENCE OF RECENT ANIMAL BITE OR SCRATCH

**Ariela Alonso, DO (Resident)**; Leonie Deniau, (Medical Student); Latha Kannan, MD (Faculty).

SCS-affiliated Resident Physician or Fellow; Detroit Wayne County Health Authority, MI.

### BACKGROUND

Spontaneous bacterial peritonitis (SBP) is an infection of intra-abdominal fluid that is seen in patients with advanced liver or kidney disease. It occurs in 10% to 30% of patients receiving multiple paracentesis for ascites or on dialysis. Enteric microorganisms are the most common cause of SBP. Pneumatosis Coli is the presence of gas in the intestinal wall mainly caused by aerobic organisms. *Pasteurella multocida* is a facultative anaerobic gram-negative bacilli organism most commonly causing soft tissue infections caused by a bite or scratch from dogs and cats. However, other infections with this organism tend to be rare. There has been an increase in cases of SBP being caused by *Pasteurella multocida* without evidence of an animal bite or scratch.

### CASE DESCRIPTION

A 61-year-old African American male with a past medical history of HIV, end stage renal disease on hemodialysis, hypertension, COPD, systolic heart failure, recurrent ascites, chronic thrombocytopenia, cirrhosis, and hepatitis C presented to the ED for worsening abdominal pain with tachy- cardia and fever during his dialysis session. Physical examination revealed tachycardia, distended, diffusely tender abdomen with positive fluid thrill and no skin lesions, open wounds, or rashes appreciated. Labs were obtained, Lactic acid 3, WBC 5.3, Platelets 58, 1 out of 2 blood cultures positive for gram-positive rods. Started on Rocephin and Flagyl. Paracentesis was performed and 5L of clear yellow ascitic fluid was removed. CT abdomen findings revealed pneumatosis coli of the ascending colon and cecum and diffuse peritoneal thickening suspicious for peritonitis. US abdomen revealed cirrhotic liver. Fluid culture was positive for *Pasteurella multocida* with WBC 3278 and blood culture grew *Pasteurella multocida*. Antibiotics were changed to Unasyn. Repeat CT Abdomen revealed the patient no longer had pneumatosis coli.

### CONCLUSION

It is likely that the infection resulted from a domestic cat, as the patient reported having cats in his home. Being immunocompromised and non-compliant with follow up and medications puts him at a higher risk of infection without a recent animal bite or scratch. Given the rarity of SBP with Pasteurella, few reports have discussed this condition and clinical overlap of alternative bacterial causes.

Email of Presenting Author: Aalonso-gme@authority-health.org

## 18

## SURROGATE MARKERS FOR TOXIC ALCOHOL INGESTION GUIDE MANAGEMENT: A CASE REPORT OF ACCIDENTAL ETHYLENE GLYCOL INTOXICATION

**Ashish Tripathi, BS (Medical Student)**; Anthony Teta, MD; Christopher Provenzano, MD (Faculty).

MSUCOM Medical Student; MSUCOM.

### BACKGROUND

Ethylene glycol poisoning is a rare, life-threatening emergency that requires early detection and rapid treatment. Several clinical signs and markers suggest ethylene glycol intoxication; however, quantitative assessment of exposure by gas chromatography is limited by its delays.

### CASE DESCRIPTION

A 56-year-old woman presented unresponsive with an un- known downtime. Her past medical history is noncontributory. She had a severe high anion gap metabolic acidosis (27 mmol/L), an elevated osmolar gap (17 mOsm/kg), creatinine of 1.98 mg/dL, blood urea nitrogen of 23 mg/dL and estimated glomerular filtration rate of 28 mL/min. Volatile screening returned negative, and serum ethylene glycol levels were pending. She was started on fomepizole, a sodium bicarbonate infusion, and underwent emergent hemodialysis for possible ethylene glycol ingestion. Serum ethylene glycol level returned on hospital day two and was unremarkable. A kidney biopsy revealed diffuse acute tubular injury with oxalate deposition in the tubular lumens, consistent with oxalate nephropathy secondary to ethylene glycol ingestion. Further investigation revealed she had been drinking water from a winterized mobile home. She was discharged with outpatient dialysis. At one year follow- up, her kidney function has returned to baseline.

### DISCUSSION/CONCLUSION

Serum levels of ethylene glycol peak between one- and four-hours following ingestion; however, ethylene glycol under- goes rapid metabolism, and serum levels can be undetectable if a patient presents later in their course. The gold standard gas chromatography isn’t widely available, and results are usually delayed. Clinicians should initiate treatment for ethylene glycol poisoning based on surrogate markers of toxicity.

Email of Presenting Author: tripat16@msu

## 19

## PERSISTENT HEART FAILURE DESPITE MEDICAL THERAPY, LEADING TO A DIAGNOSIS OF CARDIAC AMYLOIDOSIS

**Diva Maraj, MD (Resident)**; Parth Patel, MD (Resident); Sruthi Ramanan, MD (Resident); Mumtaz Memon, MD, FACP (Faculty); Elise Hawes, BAS, ACS, RDCS, FASE (Faculty).

SCS-affiliated Resident Physician or Fellow; Henry Ford Jackson, MI.

### BACKGROUND

Cardiac amyloidosis is a restrictive cardiomyopathy, de- fined as either light chain amyloidosis (AL) or transthyretin amyloidosis (ATTR), which can be further subdivided into wild-type (systemic senile amyloidosis) and hereditary transthyretin amyloidosis. Amyloidosis has a poor prognosis with a survival rate of six months and up to five years.

### CASE DESCRIPTION

We present a 72-year-old female with a past medical his- tory of heart failure, atrial fibrillation, systemic lupus erythematosus (SLE), and Stage 3b chronic kidney disease, who presented with persistent shortness of breath, lower extremity pitting edema, jugular venous distension, and dyspnea despite optimal medical therapy. The patient was started on furosemide, 80 mg intravenously, with only a small improvement in her symptoms. The patient’s previous echocardiogram revealed an ejection fraction of 25-30%. Due to a poor response to diuretics, the patient underwent a cardiac MRI (CMR), monoclonal gammopathy testing, and a Technetium pyrophosphate (99mTc-PYP) scintigraphy, of which the latter two were unrevealing. The CMR revealed a left ventricular ejection fraction of 52%, with increased wall thickness and multiple segments of mid myocardial to subendocardial late gadolinium enhancement, suggestive of an infiltrative disease. Due to inconclusive testing, the patient underwent an endomyocardial biopsy and was determined to have wild-type, systemic senile amyloidosis. The patient was started on tafamidis, a new Food and Drug Administration (FDA) approved therapy for systemic senile amyloidosis and was discharged on the new medication, with frequent follow-up visits scheduled.

### DISCUSSION/CONCLUSION

Current treatment guidelines for cardiac amyloidosis include loop diuretics as well as spironolactone. Medications such as beta blockers, angiotensin converting enzyme inhibitors and calcium channel blockers are not clinically effective. There are currently new medications on the horizon, such as tafamidis, which stabilizes the transthyretin tetramer and reduces the formation of amyloid. This case highlighted that patients who have persistent symptoms of heart failure, despite guideline-directed medical therapy, and without a history of genetic cardiac conditions, may also have a diagnosis of cardiac amyloidosis. Cardiac amyloidosis is often misdiagnosed or diagnosed late in the dis- ease course; therefore, there is a need for increasing aware- ness of early diagnosis and treatment, including new FDA-approved medications for a better chance of survival.

Email of Presenting Author: chalisamaraj@gmail.com

## 20

## ENDOSCOPIC CHOLANGIOSCOPY WITH CHOLELITHIASIS LITHOTRIPSY USING MINIMALLY INVASIVE PERCUTANEOUS (MIPS) TRILOGY LITHOTRIPTER: A CASE REPORT

**Gabriela Mazur, DO (Resident)**; Anthony Bonzagni, DO; Harris Shaikh, DO; Shehbaz Shaikh, MD; Robert Elgin, DO (Faculty).

SCS-affiliated Resident Physician or Fellow; Urology Residency- McLaren Macomb, MI.

### BACKGROUND

Acute cholecystitis affects 200,000 people each year in the US. Percutaneous cholecystostomy tube is an effective therapy for acute cholecystitis for patients with high perioperative risks; however, it carries higher rates of postprocedural complications compared to laparoscopic cholecystectomy. Previous reports using percutaneous nephrolithotripter are reported with 24- to 30-French access sheaths to clear gallstone burden. There are no reports of using percutaneous minimally invasive access with micro-lithotripter to treat cholelithiasis percutaneously.

### CASE DESCRIPTION

A 57-year-old female with chronic cholecystitis and numerous comorbidities preventing safe cholecystectomy has been treated with chronic 16-French cholecystostomy tube. Previous attempt at clearing gallstones using Spyglass cholangioscopy was unsuccessful. We performed minimally invasive access lithotripsy using micro-Trilogy lithotripter with complete clearance of > 5cm stone burden. To begin, an occlusion balloon was placed by interventional radiology in the cystic duct to prevent 0.9% normal saline irrigant to leak into small bowel. Transhepatic dilation of tract to 16.5fr was achieved using Storz MIP-S set. Total fragmentation time was 3.5 hours with no remaining stone fragments upon completion. Patient underwent successful choleystostomy tube removal two weeks later.

### CONCLUSION

A successful use of mini lithotripter has been demonstrated for clearance of high burden of cholelithiasis. This may be a viable and safer option for gallstone clearance in otherwise nonsurgical candidates.

Email of Presenting Author: gabriela.mazur@mclaren.org

## 21

## BIO-INDUCTIVE COLLAGEN IMPLANT AUGMENTATION FOR MYOTENDINOUS ACHILLES RUPTURE IN A TEENAGE COMPETITIVE GYMNAST: A CASE REPORT

**Josiah Valk, DO (Resident)**; Michael J Wilk, DO (Resident); Kelly Murdock, PA-C (Physician Assistant); Mohamed Saad, MD (Faculty).

SCS-affiliated Resident Physician or Fellow; Corewell Health System, MI.

Achilles tendon ruptures are common in the 4th or 5th decade of life, with a strong male predilection. Consequentially, female pediatric Achilles tendon ruptures are exceedingly rare and published reports are limited to case series. These injuries may be devastating for competitive athletes with a return to sport rate of only 70-76% in professional athletes. There is no consensus on optimal management for these injuries. Treatment options include nonoperative management with immobilization and functional rehabilitation whereas surgical options include open versus per- cutaneous primary repair. Ruptures at the myotendinous junction represent particularly challenging repairs as the muscular proximal limb has less ability to hold suture than the tendinous distal limb. In rotator cuff surgery, bio-inductive collagen implants have been safe and effective, demonstrating the ability to induce the growth of tendon-like tis- sue with increased tendon thickness maintained for several years. Bio-inductive implants are not yet well studied in the augmentation of Achilles tendon repairs, but active trials are ongoing. We present the rare case of a traumatic Achilles tendon rupture at the myotendinous junction of a 16-year-old elite gymnast treated with open direct repair with bio-inductive patch augmentation. The patient had excellent functional results and is back to competitive sport at 1-year follow-up and demonstrated increased tendon thickness on MRI (magnetic resonance imaging).

Email of Presenting Author: Josiahvalk@gmail.com

## 22

## A CASE OF RECURRENT LEFT-SIDED HEPATIC HYDROTHORAX IN A CHRONICALLY DEBILITATED PATIENT: UNCOMMON PRESENTATION

**Khaled M. Harmouch, MD (Resident)**; Hassan M.A. Makki, MD, FCCP (Faculty).

SCS-affiliated Resident Physician or Fellow; Detroit Medical Center-Sinai Grace Hospital, MI.

### INTRODUCTION

Hepatic hydrothorax [HH] is an excessive accumulation of >500 ml of transudative fluid in the pleural cavity of patients with decompensated liver cirrhosis. HH is not frequently seen and constitutes only 2-3% of all causes of pleural effusion. 85% of cases are right-sided, 13% are left- sided, and 2% have fluid in the pleural cavity on both sides. We describe a case of recurrent left-sided transudative pleural effusion in a patient with alcoholic liver cirrhosis and ascites.

### CASE DESCRIPTION

A 47-year-old quadriplegic female with a past medical history of chronic hypoxic respiratory failure S/P tracheostomy and liver cirrhosis with ascites and hepatic encephalopathy was admitted to the hospital for a clogged nasogastric tube. Her vital signs were blood pressure of 153/94 mmHg, heart rate of 133 beats/min, respiratory rate of 33 cycles/min, SPO2 of 96% on room air, and temperature of 39.5°C. Physical examination revealed mild abdominal distension and bilateral pedal edema. Laboratory evaluation was notable for anemia with hemoglobin 7.6g/dL, leukocytosis 17,000/mm3, total bilirubin 1.2 mg/dL, PT 14.8 s, and INR 1.4. CT abdomen & pelvis with contrast showed cirrhotic liver and moderate ascites. The patient was noted to have increased tracheostomy secretions after which she developed respiratory distress and required mechanical ventilation. CT-PE revealed pulmonary emboli in the right lobe and pleural effusion in the left lobe. Her hospital stay was further complicated by hospital-acquired pneumonia. CXR revealed left-sided moderate pleural effusion. Thoracentesis was performed and 1250 clear yellow fluid was removed. Fluid analysis revealed a pleural LDH of 32, pleural protein of 3 serum protein of 6.3, and serum LDH of 249, consistent with transudative effusion. Follow-up imaging revealed recurrent left-sided pleural effusion that required multiple thoracocenteses.

### CONCLUSION

Hydrothorax is a rare complication of liver cirrhosis that predominantly occurs on the right side but may also develop on the left side. Left-sided hydrothorax might be as- sociated with a high risk of recurrence in which repeated thoracocentesis is potentially required. Diagnostic thoracentesis is important to rule out other etiologies of pleural effusions in this category of patients.

Email of Presenting Author: kharmouc@dmc.org

## 23

## AN UNEXPECTED POST-PARTUM COMPLICATION OF HEMORRHAGIC STROKE

**Lucy Bolerjack, DO (Resident)**; Sarah Hopkins, DO (Resident); Andrew Victor, MD (Resident); Jennifer DeAnna, DO (Faculty); Dylan Mooney, DO (Faculty).

SCS-affiliated Resident Physician or Fellow; Ascension Genesys, MI.

### CASE DESCRIPTION

A 34-year-old G6P4 female who was six days postpartum from an uncomplicated vaginal delivery presented to Labor & Delivery triage with complaints of severe headache and neck pain. Her initial blood pressures ranged from 160-200 mmHg systolic and therefore under suspicion for preeclampsia with severe features, intravenous Hydralazine and Magnesium were initiated. While being assessed in triage, the patient was observed by nursing staff to have a single moment of “spasm” which was followed by acute mental status change of somnolence with left sided facial droop. Code stroke protocol was immediately initiated, and the patient was found to have an intracerebral hemorrhage in the left caudate nucleus basal ganglia area on a non-contrast CT scan of her brain. Neurosurgery was emergently consulted and a repeat CT venogram ruled out sinus thrombosis as well as evidence of aneurysm. The repeat CT confirmed the bleed to be stable however had ruptured into the ventricle system. Throughout this time, the patient’s neurological symptoms continued to progress and deteriorate including her left sided facial droop, new dysarthria and right sided upper and lower extremity weakness.

The patient was started on a Nicardipine drip and given a Levetiracetam loading dose before being emergently transferred to another facility for neurotrauma intensive care and monitoring. In the setting of severe range blood pressures, the patient has an atypical presentation of eclampsia as there was no history of preeclampsia during pregnancy nor was there evidence of this in her admission blood work which included an unremarkable CBC, chemistry panel, liver function testing, protein/creatinine ratio, urinalysis and urine drug screen.

## DISCUSSION/CONCLUSION

Per literature review, 20% of eclampsia occurs post-partum and of those, 90% occur within one week of delivery. The most common presenting symptom is headache occurring in 70% of cases. This case highlights spontaneous cerebral hemorrhage secondary to elevated blood pressures and eclampsia in an otherwise healthy 34-year-old female with no significant past medical history including chronic hypertension, gestational hypertension or preeclampsia in previous pregnancies.

Email of Presenting Author: lucy.bolerjack@ascension.org

## 24

## AN UNUSUAL PRESENTATION OF A RARE PHENOMENON: HEMORRHAGIC CHOLECYSTITIS IN THE SETTING OF UNEXPLAINED ANEMIA

**Rachel Cohen, DO (Resident)**; Dillon Jarbo, MD (Resi- dent); Devan Schlund, MD (Faculty).

SCS-affiliated Resident Physician or Fellow; Ascension Macomb-Oakland Hospital, MI.

### BACKGROUND

While acute cholecystitis represents a common presentation in the ED, hemorrhagic cholecystitis represents a rare but possibly fatal complication of gallbladder disease. As- sociated risk factors include anticoagulation therapy, trauma, malignancy, coagulopathy, renal failure, and cirrhosis. Hemobilia may present with Quincke’s triad of RUQ pain, jaundice, and GI bleeding in up to 22% of patients and can be seen in patients with acute hemorrhagic cholecystitis, though none of these symptoms were noted in this case.

### CASE DESCRIPTION

We present a case of a 65-year-old man on dual-antiplatelet therapy who initially presented with constipation for 3 weeks. He became encephalopathic on hospital day #2 due to a septic joint and was taken for knee washout with the Orthopedic Surgery team. He was noted to have steadily worsening anemia throughout his hospital course resulting in blood transfusion requirement on hospital day #9 in the absence of any obvious signs of melena, hematochezia, or other bleeding. At that time, a CT angiogram of the abdomen/pelvis was obtained which showed a mixed density in the gallbladder lumen with areas of intraluminal hemorrhage consistent with hemorrhagic cholecystitis without other suspicious signs of intra-abdominal bleeding elsewhere. At time of surgical evaluation, the patient had not had abdominal pain for several days prior but was taken for a laparoscopic fenestrating subtotal cholecystectomy mere hours after surgical evaluation. Intraoperatively, he was noted to have a large amount of clot within the lumen of the gallbladder as well as a 2.9 cm pigmented stone.

### CONCLUSION

Hemorrhagic cholecystitis represents a rare complication of acute cholecystitis and is relegated to mostly case reports in scientific literature. In the setting of unexplained anemia, it should remain on the differential as a source of occult GI bleeding. Failure to recognize this disease process could result in gallbladder rupture, hemorrhagic shock, or even death.

Email of Presenting Author: rachel.cohen@ascension.org

## 25

## A RARE CASE OF GASTROINTESTINAL VASCULITIS CONFIRMED BY RADIOLOGICAL MODALITY

**Rashona A. Moss, DO, (Resident)**; Shurook Elassouli, MD; Sujata Kambhatla, MD (Faculty).

SCS-affiliated Resident Physician or Fellow; Garden City Hospital, MI.

### BACKGROUND

Vasculitis is inflammation of blood vessel walls with re- active damage to mural structures. 20% of systemic vasculitis cases have GI manifestations, however, isolated vasculitis of GI tract is rare. In this case, the patient presented with abdominal pain with elevated inflammatory markers and imaging suggestive of gastrointestinal vasculitis, with negative antibodies.

### CASE DESCRIPTION

We report a case of a 62-year-old male that presented with epigastric and left upper quadrant abdominal pain with nausea and vomiting for one day. He has a past medical history of diabetes mellitus type II, hypertension, hyperlipidemia, and polycythemia. He denied any fever, rash, arthralgias, weight loss or hematemesis. Physical exam findings include tenderness to palpation to epigastric area, left upper quadrant and left flank. Patient remained hemodynamically stable throughout his admission. Computed tomography (CT) abdomen and pelvis showed splenic infarction with abnormal inflammation of celiac, common hepatic, and proximal splenic arteries suggestive of vasculitis. CT angiogram (CTA) abdomen and pelvis con- firmed the same findings and also showed dissection of distal splenic artery extending into proximal splenic artery with a non-occlusive thrombus in proximal left splenic artery.

Based on CT abdomen findings, further workup for systemic vasculitis revealed elevated erythrocyte sedimentation rate (ESR), c-reactive protein (CRP), alkaline phosphatase, hemoglobin 16.7, and hematocrit 50.9, but showed negative antibodies.

He was treated with pulse dose methylprednisolone 1 gram intravenously daily for 3 days and heparin drip for splenic artery thrombosis. His abdominal pain improved, and he was discharged on prednisone and anticoagulation. One week after discharge, abdominal pain worsened, and he was readmitted to the hospital. Repeat CTA redemonstrated splenic infarcts, but there was no documented inflammation suggestive of vasculitis. Inflammatory markers showed a significant decrease with CRP and ESR and additional rheumatological workup for vasculitis was negative.

### CONCLUSION

Isolated gastrointestinal vasculitis is rare and requires correlation between clinical, laboratory and radiological findings (1) to establish a diagnosis and start treatment in a timely manner. In our case, GI vasculitis was diagnosed by radiological findings and elevated inflammatory markers, with no signs of systemic vasculitis. Close follow-up of these patients is required to look for potential systemic involvement in future.

Email of Presenting Author: rashonamoss@gmail.com

## 26

## CHRONIC PULMONARY ASPERGILLOSIS IN AN IMMUNOCOMPETENT PATIENT

**Sahil Sethi (Medical Student)**; Ahmad Al-Sahli, MD (Resident).

MSUCOM Medical Student; MSUCOM, Ascension Macomb-Oakland, MI.

### BACKGROUND

Chronic Pulmonary Aspergillosis is a rare form of pneumonia with significant risk factors, including prolonged neutropenia, solid organ transplantation, and chronic granulomatous disease (CGD). However, we present a patient who developed invasive aspergillosis with adequate WBC counts and no history of immunocompromisation.

### CASE DESCRIPTION

The patient is a 42-year-old female with asthma on albuterol as needed who presented to the ED with fever, shortness of breath, pleuritic chest pain, and green productive sputum for 2 weeks. The patient was recently in the ER for similar symptoms and sent home with a 10-day course of doxycycline before returning 5 days later with worsening symptoms. The patient was admitted for failed outpatient community acquired pneumonia (CAP). CXR on admission revealed worsening diffuse patchy infiltrates bi- laterally when compared to previous CXR in the ED a week prior. She became hypoxic, requiring 2L of oxygen. The patient was started on IV Ceftriaxone and Azithromycin, but saw minimal clinical improvement, and was placed on IV Zosyn. She was also briefly started on IV Vancomycin, but discontinued after MRSA screening came back negative. She was sent for a CT Thorax with contrast, revealing bi- lateral cavitary pulmonary lesions with surrounding consolidation, concerning for necrotizing pneumonia. The patient was found to be HIV, ANA negative, and ACE levels were normal. Legionella, CMV, Mycoplasma and Chlamydia pneumonia came back negative. A Bronchoscopy with Endobronchial Ultrasound (EBUS) and Trans needle biopsy aspiration was performed, revealing copious, mucopurulent, yellow secretions throughout the tracheobronchial tree. A Bronchoalveolar lavage (BAL) was also performed in the RLL superior segment (B6) and sent for bacterial, AFB, fun- gal and viral analysis. Fungal cultures came back positive for Aspergillus Fumigatus. After completing 6 days of IV Zosyn and significant clinical improvement, patient was scheduled for discharge on IV Invanz for 2 weeks and oral voriconazole for 12+ weeks.

### CONCLUSION

Previous studies have found that significant risk factors include a prolonged, severe neutropenic state, HIV, or his- tory of stem cell/solid organ transplant. We propose this patient’s environmental conditions contain mold, and the patient unknowingly inhaled it for years, allowing it to by- pass normal bronchial defense mechanisms.

Email of Presenting Author: sethisah@msu.edu

## 27

## AN UNUSUAL CASE OF ABO HEMOLYTIC DISEASE INVOLVING MATERNAL BLOOD TYPE A AND NEONATAL BLOOD TYPE AB

**Sandeep Bhatti, DO (Resident)**; Prasiksha Sitaula, MD; Kanta Bhambhani, MD; Bulent Ozgonenel, MD (Faculty).

SCS-affiliated Resident Physician or Fellow; Authority Health, MI.

### BACKGROUND

ABO hemolytic disease is a common cause of hemolytic disease in the newborn, which almost exclusively occurs in babies with type A or B blood group born to mothers with type O blood group. Anti A, B antibody which is an IgG antibody present in mother with type O blood group is considered culpable for this. Hemolytic disease in newborn with ABO incompatibility, maternal blood A or B, has not been reported. Here, we report a case of neonatal hemolytic disease occurring in a baby with type AB blood group born to a mother with type A blood group.

### CASE DESCRIPTION

A male baby was born from a second pregnancy at 36 weeks 4 days via spontaneous vaginal delivery with a birth weight of 3,100 gm. The baby developed hyperbilirubinemia (Total bilirubin level of 9.18 mg/dl and direct bilirubin of 0.59 mg/dl at 15 hours of life), hemoglobin level of 16.1 g/ dl, hematocrit 45 % and 6.6 % reticulocytes. The peripheral blood smear showed occasional spherocytes. Cord blood Direct Antiglobulin Test (DAT) was positive and Anti-B anti- bodies were detected in the cord blood. Maternal blood group was A positive and the infant’s blood group was AB positive. Maternal indirect antibody test was negative. The patient received phototherapy for 28 hours until the serum total bilirubin level dropped down to 10 gm/dl. The baby was stable and maintained normal bilirubin and blood counts without evidence of hemolysis at 4 weeks of age.

### DISCUSSION/CONCLUSION

Hemolytic disease in newborn due to ABO incompatibility in mothers with blood group O occurs due to the presence of anti-A, B antibody which is an IgG type of antibody and can easily cross the placenta. In type A blood group, the serum contains anti B antibody, IgM type of antibody and cannot cross the placenta. Our case is an exception as maternal anti B antibody crossed the placental barrier and caused significant hemolysis in the newborn requiring treatment. In our literature search, till date there has not been any case reported with such unusual. ABO hemolytic disease secondary to incompatibility between maternal A and AB blood group in the newborn.

Email of Presenting Author: sandyb-hatti809@gmail.com

## 28

## LIP SWELLING AS SOLE PRESENTING SYMPTOM IN 4-YEAR-OLD FEMALE FOUND TO HAVE CROHN’S DISEASE

**Sushmitha Meghashyam, MD (Resident)**; Sandeep Bhatti, DO (Faculty).

SCS-affiliated Resident Physician or Fellow; Authority Health, MI.

### BACKGROUND

Inflammatory Bowel Disease is a group of idiopathic chronic inflammatory gastrointestinal conditions, with ulcerative colitis (UC) and Crohn’s disease being the two main disease categories. Crohn’s disease can affect any segment of the gastrointestinal tract from the oral cavity to the anus. While the gastrointestinal inflammation leads to abdominal pain, cramping, diarrhea, tenesmus, and weight loss among others being the common presenting features of both UC and Crohn’s disease, involvement of the oral cavity is not unusual in a patient with Crohn’s disease. Without the common gastrointestinal signs of inflammation, it is easy to miss out on Crohn’s disease. Here we present the case of a 4-year-old girl presenting with repeated episodes of lip swelling, leading to a diagnosis of Crohn’s disease.

### CASE DESCRIPTION

A 4-year-old female with eczema presents with two months of upper lip swelling, yellow honey crusted lesions, bleeding with friability and significant pain making it difficult to open her mouth leading to poor oral intake. She was started on IV Clindamycin for lip cellulitis with impetigo. In the hospital, she developed a rash that progressed to discrete erythematous tender nodules on b/l lower extremities consistent with erythema nodosum, raising suspicion for underlying autoimmune pathology. Blood work showed elevated CRP at 49.5 and elevated fecal calprotectin at 756. Upper and lower GI endoscopy showed scattered ulcerations with friable tissue throughout sigmoid and transverse colon. Colonic tissue biopsies demonstrated areas of chronic and focal active colitis with non-necrotizing granulomas, crypt abscesses, and lip tissue samples showed non- necrotizing granulomas, confirming the diagnosis of Crohn’s disease.

### DISCUSSION/CONCLUSION

There has been a global increase in the incidence of Crohn’s disease in the pediatric population. The clinical presentation of orofacial granulomatosis is highly variable but recurrent facial swelling, especially the lips with or without intraoral manifestation, is the most common clinical sign at onset. In the pediatric population orofacial granulomatosis may frequently represent as an extra-intestinal manifestation of Crohn’s disease and oral lesions can be the first sign of the disease. Therefore, it is important to be vigilant to identify patients and ensure time-sensitive interventions to manage and prevent disease progression.

Email of Presenting Author: smeghash@gmail.com

## 29

## ATYPICAL PRESENTATION OF ACUTE HEMORRHAGIC CHOLECYSTITIS

**Shamaiza Waqas, MD (Resident)**; Stephen Kehres, DO (Faculty); Waqas Abid, MD; Matthew Stander, DO.

SCS-affiliated Resident Physician or Fellow; Ascension Macomb-Oakland Hospital, MI.

### BACKGROUND

Hemorrhagic cholecystitis is rare complication of acute cholecystitis. Acute hemorrhagic cholecystitis can be associated with a high mortality rate of up to 20%. Symptoms of acute hemorrhagic cholecystitis can mimic those of acute cholecystitis such as right upper quadrant pain, fever, and leukocytosis. Rare presentations include obstructive jaundice, cholangitis from the clot resulting in CBD obstruction, hematemesis and or melena. Typical presentation of acute hemorrhagic cholecystitis is only seen in less than 25% of patients presenting with this pathology.

### CASE DESCRIPTION

A 64-year-old Caucasian male with past medical history of NIDDM, CAD, HTN and chronic osteomyelitis of foot, presented with Altered Mental Status likely related to acute foot infection. Patient was started on IV antibiotics and had amputation of foot. Patient’s symptoms of acute encephalopathy subsequently resolved. During the post operative stay in the hospital, his hemoglobin started to decline without any obvious source of bleeding. Patient did not complain of nausea, vomiting or abdominal pain. Patient’s abdomen was found to be soft, non-tender and non-distended. Subsequently he became vitally unstable. Pertinent lab work showed acute anemia with Hb of 5.6 gm/dL, leukocytosis (WBC of 11.49 k/mcL), thrombocytosis (platelets of 441 k/mcL) and elevated ALP (188 IUnits/L). AST and ALT were within normal limits. CT angiogram was ordered to rule out acute gastrointestinal bleed and showed findings related to acute hemorrhagic cholecystic with a distended gallbladder, gallstone, and hemorrhage within the gallbladder lumen. Patient subsequently had emergent laparoscopic cholecystectomy and was latter discharged home in a stable condition.

### CONCLUSION

Acute hemorrhagic cholecystitis is a rare complication of acute cholecystitis with usually an atypical presentation and therefore early and prompt diagnosis and management remain challenging for clinicians. Acute Hemorrhagic cholecystitis should be considered as a crucial differential diagnosis in patients with acute blood loss anemia with no obvious source of bleeding and misleading negative physical exam findings especially in critically ill patients to re- duce the associated high mortality. More awareness regarding this pathology and its atypical presentation can help clinicians be better suited to investigate for this pathology and lead to early diagnosis and prompt treatment leading to better patient outcome.

Email of Presenting Author: shamaiza.waqas@ascension.org

## 30

## BLADDER CANCER CAUSING BLADDER PERFORATION, RESULTING IN EMERGENT CYSTOPROSTECTOMY WITH ILEAL LOOP DIVERSION

**Upahvan Rai, DO (Resident)**; Brandon Tompkins, DO (Resident); Seth Thomas, DO; Ali Rizvi, BS; Richard Sarle, MD (Faculty).

SCS-affiliated Resident Physician or Fellow; Sparrow Hospital, MI.

### INTRODUCTION

Cystoprostatectomy with ileal loop diversion is an established treatment option for bladder cancer, especially muscle invasive bladder cancer, but its role in the emergent setting is not well documented. There have been reported cases of spontaneous bladder perforation due to bladder cancer, which presents as an acute abdomen. A number of these patients underwent primary closure of the perforation. Unfortunately, outcomes were poor. Of note, the mortality rate from spontaneous bladder rupture is 25-47% and 80% if associated with an undiagnosed bladder tumor.

### METHODS

A PubMed search was conducted for emergent cystoprostatectomy due to a perforated bladder mass. There are a handful of case reports discussing this topic, in addition to a single center retrospective study reviewing bladder perforation during transurethral resection of non-muscle invasive bladder cancer. But there is no established study dis- cussing emergency cystoprostatectomy due to high grade muscle invasive bladder cancer causing bladder perforation.

### RESULTS

A 75-year-old male with a significant smoking history and no past medical history presented with recent falls and new onset gross hematuria. CT imaging showed a large bladder mass and left sided hydronephrosis. Intra-op, a large bladder perforation and bladder mass was found. An emergent cystoprostatectomy with ileal loop diversion was per- formed. Pathology showed high grade muscle invasive bladder cancer with necrosis and perforation.

### CONCLUSION

Our case report, although uncommon, shows advanced bladder cancer with perforation at presentation, requiring emergent cystoprostatectomy. Furthermore, it illustrates perforation is a possibility with bladder masses, and having a well-established plan prior to any intervention is para- mount.

Email of Presenting Author: u_rai0128@email.campbell.edu

## 31

## TEXTURE ANALYSIS OF FEMORAL CARTILAGE ULTRASOUND HIGHLIGHTS SUBTLE BETWEEN LIMB DIFFERENCES IN CARTILAGE HEALTH IN PATIENTS AFTER ACL RECONSTRUCTION

**Anthony L. Lewis (Medical Student)**; Matthew S. Harkey, PhD, ATC (Faculty).

MSUCOM Medical Student; MSUCOM.

### BACKGROUND

Monitoring femoral articular cartilage morphology alterations in patients after ACL reconstruction (ACLR) may be an effective way to identify patients at high risk for developing knee osteoarthritis. Diagnostic ultrasound is a clinically valid imaging modality for assessing femoral cartilage morphology. Degenerative femoral cartilage alterations are reflected by changes in spatial distribution of pixel values and are therefore quantifiable by gray level co-occurrence matrix (GLCM) texture analysis. We hypothesized cartilage thickness, echo-intensity, and GLCM texture metrics can detect alterations in femoral articular cartilage when com- paring a patient’s ACLR limb to their contralateral limb.

### METHODS

Twenty-two individuals with a history of unilateral ACLR within the prior 4-7 months participated in this study. A single investigator used a transverse suprapatellar ultra- sound scan to obtain bilateral images of the femoral cartilage. We used our semi-automated cartilage segmentation program to segment the femoral cartilage cross-sectional area. After segmentation, the program automatically calculated the following variables: 1) average cartilage thick- ness, 2) average echo-intensity, 3) GLCM texture metrics (i.e., contrast, correlation, energy, homogeneity). The cartilage thickness, echo-intensity, and GLCM texture metrics were averaged across three images acquired in the ACLR and contralateral limb for each participant. Dependent t- tests were used to compare the ultrasound outcomes be- tween the ACLR and contralateral limb.

### RESULTS

There were not significant differences between limbs for the traditional cartilage morphology variables of average cartilage thickness (t=1.20, p=0.24) and echo-intensity (t=-0.78, p=0.44). There was a significantly moderate difference in GLCM correlation between the ACLR and contralateral limb, with ACLR correlation being less (t=-2.21, p=0.04; d:-0.47). However, there were no other significant between limb differences for the other GLCM texture metrics (p-value range: 0.18–0.25).

### CONCLUSIONS

These results indicate that cartilage texture analysis metrics may be more sensitive than traditional cartilage morphology metrics to identifying differences between limbs in patients following ACLR. Specifically, the ACLR cartilage has a lower joint probability occurrence for specified pixel value pairs compared to the contralateral limb. Further re- search is required to determine the clinical importance of early cartilage alterations following ACLR and if administering OA prevention interventions may be able to improve cartilage metrics in these patients.

Email of Presenting Author: lewisa52@msu.edu

## 32

## HOSPITAL OUTCOMES FOR ICU PATIENTS WITH SPUTUM POSITIVE SEVERE COMMUNITY- ACQUIRED PNEUMONIA

Brandon Govan, MD (Resident).

SCS-affiliated Resident Physician or Fellow; Ascension Genesys, MI.

### BACKGROUND

Community-acquired pneumonia (CAP) is one of the most common infections worldwide. In the United States it causes greater than 1.5 million hospitalizations and ap- proximately 100,000 deaths/year. Sputum cultures are a common diagnostic study used to evaluate CAP. American Thoracic Society (ATS) guidelines recommend that sputum cultures are performed for patients with severe CAP despite mixed/poor evidence that cultures improve outcomes. Some studies comment that their low diagnostic yield limits their impact on antibiotic de-escalation and mortality. Others report decreased hospital length of stay (LOS) and duration of IV antibiotics secondary to targeted therapy. Due to the continued controversy surrounding sputum cultures this study looked to determine if patients admitted to the intensive care unit (ICU) with severe CAP and positive sputum culture had improved hospital outcomes.

### METHODS

A single center retrospective chart review of patients admitted to the ICU with CAP from January 2013 to December 2018 was conducted. Infectious Diseases Society of America/ATS criteria identified patients with severe CAP. Pneumonia severity index and hospital outcomes (IV antibiotic days, ICU/hospital LOS, ventilator days, mortality) were recorded. Three groups (sputum positive, sputum negative, sputum not obtained) were compared using ANOVA with Bonferroni post hoc analysis.

### RESULTS

There were 206 patients evaluated. The mean age was 72.0 (SD: 13.1) years old and the majority were white (93.7%). Sputum culture was obtained in 62.6% (n=129) of patients, 29.5% (n=38) had a positive result. There was a significant difference in mean IV antibiotic days between the positive and no culture groups (9.3 vs 5.2 days, p=0.001), and the negative and no culture groups (8.9 vs 5.2 days, p<0.0001). Hospital LOS was significantly longer for patients who had a positive sputum culture versus no culture (10.2 vs 5.6 days, p<0.0001), and for those with a negative culture versus no culture (9.9 vs 5.6 days, p<0.0001). No significant difference in mortality was seen between groups (p=0.93).

### CONCLUSIONS

Patients with severe CAP who had a sputum culture (positive or negative) had a longer duration of IV antibiotics and hospital LOS compared to patients who did not have a culture. Mortality did not differ between groups.

Email of Presenting Author: brandon.govan@ascension.org

## 33

## THE ENIGMA OF EOSINOPHILIC ESOPHAGITIS AND PERIPHERAL EOSINOPHILIA

**Brandon Wiggins, DO, MPH (Resident)**; Reginald Sandy, DO (Faculty).

SCS-affiliated Resident Physician or Fellow; Ascension Genesys, MI.

### BACKGROUND

Eosinophilic Esophagitis (EoE) is a fairly new disorder of the gastrointestinal tract, first identified in the 1990’s. Typically, the esophagus is completely devoid of eosinophils. Previously, it was thought that gastric reflux caused an immune reaction in the esophagus, including elevation in eosinophils. Since the discovery of the entirely different entity of EoE, it was found that EoE has a significantly higher amount of eosinophils on biopsy, differing symptom prevalence (dysphagia and food impaction) and characteristic esophageal ring formation compared to GERD. Upon review of literature, very few studies have examined the relation- ship between EoE and peripheral eosinophilia measures by serology.

### METHODS

This is a retrospective study including 46 patients in a com- munity hospital setting that have been diagnosed with EoE on endoscopy and histology with eosinophil counts before diagnosis and after treatment. Paired sample t tests will be used to determine significance between eosinophil counts before diagnosis and after treatment. Observation of degree of eosinophil elevated related to diagnosis of EoE will also be performed. Patients with malignancy, parasite in- fection, previous diagnosis of EoE, antihistamine use, pro- ton pump inhibitor use and ages less than 18 or greater than 100 were excluded.

### RESULTS

Mean age of patients was 41.3 (SD 15) years old, mean length of stay was 1.3 (SD1.1) days, 27 males (58.7%) and 19 females (41.3%), 43 Caucasians (93.5%) with remaining African Americans, American Indians or Alaskan Natives, mean eosinophils 4.2 (SD 3.9%) before treatment and 2.7 (SD: 2.5%) after treatment. Based on paired samples t test there was a p value of 0.03, between eosinophil counts be- fore and after treatment.

## DISCUSSION

Based on the p value obtained via paired samples t test, there was a significant decrease in eosinophil counts after treatment. Despite this, there was not a sufficient average of eosinophils before diagnosis of EoE (6%) to confirm a relationship between peripheral eosinophilia and EoE. This study demonstrates questionable utility of screening for EoE with peripheral eosinophilia. Future studies will need to be performed to investigate the relationship, especially with a large sample size compared to this study to increase power.

Email of Presenting Author: Brandon.wiggins@ascension.org

## 34

## THE ASSOCIATION OF HIDRADENITIS SUPPURATIVA WITH TYPE 2 DIABETES MELLITUS IN BLACK/AFRICAN AMERICAN PATIENTS

**Eglal Samir, BS (Medical Student)**; Andrea Dai, BS; Rachel Krevh, BS; Ian Loveless, MS; Wan-Ting Su, PhD; Li Zhou, MD; Indra Adrianto, PhD; Qing-Sheng Mi, MD, PhD (Faculty).

MSUCOM Medical Student; MSUCOM.

### BACKGROUND

Hidradenitis suppurativa (HS) is a chronic inflammatory skin disease characterized by painful nodules, abscesses, and sinus tracts predominately in the axillary, inguinal, in- fra-mammary regions. Most studies from North America and Europe have shown that HS affects women more than men, with a female-to-male ratio of roughly 3:1. Additionally, Black/African American (Black/AA) patients have a higher HS prevalence and disease severity than White patients. Current literature also suggests a significant association between HS and several comorbidities including Type 2 diabetes mellitus (T2DM) and renal disease. Previous studies’ cohorts are limited in size and diversity, so it is still unclear if there is a significant difference in the incidence of T2DM between Black/AA and White HS patients. Furthermore, the temporal relationship is unknown between HS and T2DM. Understanding these questions will allow providers to screen for T2DM and relevant comorbidities in patients with HS, which can improve health outcomes in Black/AA patients.

### OBJECTIVE

Our aim is to investigate the relationship between HS and Type 2 diabetes comorbidities in Black/AA White patients.

### METHODS

We retrospectively analyzed 13,130 patients with HS and Type 2 diabetes in Henry Ford Health from 1995-2022.

### RESULTS

Of the 13,130 patients with HS who presented to Henry Ford Health, 54.1% were Black/African Americans and 36.3% were European American/White. After observing the overall comorbidities of patients with HS adjusted for age and sex at first diagnosis, Black/AA patients had a 1.62 (1.17-2.26) odds ratio of having diabetes with chronic com- plications compared to White patients. The odds ratio of Black/AA males having HS is 1.91 (1.24-2.89) compared to Black/AA females.

### CONCLUSION

The incidence of Type 2 diabetes mellitus with chronic complications in HS is higher in Black/AA patients, specifically in Black/AA males.

Email of Presenting Author: samiregl@msu.edu

## 35

## ARTERIAL STIFFNESS ASSESSED USING CAROTID-TO-FEMORAL PULSE WAVE VELOCITY IS UNCHANGED THROUGHOUT THE DAY IN YOUNG HEALTHY ADULTS

**Hemal Dholakia (Medical Student)**; Jill M. Slade, PhD (Faculty); Katharine D. Currie, PhD (Faculty).

MSUCOM Medical Student; MSUCOM.

### BACKGROUND

Carotid-to-femoral pulse wave velocity (cfPWV) describes the speed of the pulse in the body and is a well-established measure of arterial stiffness. A higher cfPWV value, indicative of greater arterial stiffness, is associated with cardio- vascular morbidity and mortality. While it is well established that other measures of cardiovascular health (e.g., blood pressure) demonstrate circadian rhythms and therefore vary throughout the day, less is known about daily rhythms in arterial stiffness. Understanding the impact that the time of day can have on arterial stiffness will help to in- form best practices for the measurement and interpretation of cfPWV. Therefore, the purpose of this study was to examine the effect of time of day on arterial stiffness, assessed using cfPWV. We hypothesized cfPWV would be highest in the morning compared to the other time points.

### METHODS

To date, 10 healthy adults (23±5 years; 80% female; 80% white non-Hispanic; body mass index of 22.3±1.8 kg/m2) have participated. cfPWV was measured on three separate days in the morning (0700-0900h), afternoon (1300-1500h), and evening (1800-2000h) in randomized order. Participants rested in the supine position for 10 minutes, after which pulse waves were capture with a hand-held tonometer at the carotid and femoral arterial sites and analyzed offline to determine pulse transit time. Distance between sites was measured along the surface of the body, and cf- PWV was calculated as (0.8×distance)/pulse transit time. Data were checked for normality using the Shapiro-Wilk normality test and passed. Therefore, a repeated measures analysis of variance was performed, with statistical significance set at P<0.05.

### RESULTS

There was no difference in cfPWV measured in the morning (6.4±0.7 m/s), afternoon (6.5±0.8 m/s), and evening (6.5±0.7 m/s; P=0.769).

### CONCLUSION

Our preliminary analysis demonstrates no effect of time of day on arterial stiffness assessed using cfPWV and thus there appears to be no optimal time to perform this assessment. Future studies in this area should be conducted on larger sample sizes inclusive of individuals with different demographics and health conditions.

Email of Presenting Author: dholaki9@msu.edu

## 36

## INTER-ARM BLOOD PRESSURE DIFFERENCES IN POST-MENOPAUSAL FEMALES WITH HYPERTENSION

**Hesham Atoui (Medical Student)**; Emily Ubbens (Medical Student); Kasandra Sanidad (Medical Student); Wesley T. Blumenburg, MS ; Marianne Huebner, PhD; Supratik Rayamajhi, MD; George S. Abela, MD; Jill M. Slade, PhD (Faculty); Katharine D. Currie, PhD (Faculty).

MSUCOM Medical Student; MSUCOM.

### BACKGROUND

Hypertension is currently diagnosed based on the highest measurements obtained from blood pressure (BP) readings. Differences of BP between arms (inter-arm differences or IAD) greater than 10 mmHg has been proven to increase mortality. In addition, BP has a clinically important circadian rhythm, but relatively little is known if IADs vary by time of day.

### OBJECTIVES

The purpose was 1) to examine if there are BP IADs, 2) to identify if IADs are clinically significant, and 3) to examine whether IADs vary by time of day in postmenopausal females with hypertension.

### METHODS

Post-menopausal females (ages 55-80) with hypertension had seated BPs taken bilaterally with an automated ma- chine (GE ProCare) in the morning and evening. The average of three readings between the left (L) and right arm (R) was compared using paired t-tests, with significance at p<0.05. Systolic (SBP), diastolic (DBP) and mean arterial pressure (MAP) were compared between arms; mean ±SD are reported. The IAD [R-L] was also calculated to deter- mine how many females had IAD greater than 10 mmHg.

### RESULTS

Seven participants are included in this preliminary analysis (BMI 28.3±5.5 kg/m2, 67.3±6.9 years old). Morning SBP was 152±23 (R) and 150±18 (L) mmHg (P=0.715), morning DBP was 90±15 (R) and 84±14 (L) mmHg (P=0.016), and morning MAP was 108±15 (R) and 111±15 (L) mmHg (P=0.068). The IAD was 4.7±6.2 mmHg, 6.5±4.0 mmHg and 4.8±1.6 mmHg for SBP, DBP, and MAP respectively. For SBP, one participant had an IAD >10 mmHg and one had DBP IAD >10 mmHg. Morning vs. evening IAD (n=5) was reported as follows: SBP was 7.2±7.7 (AM) and 5.4±5.5 (PM) mmHg (P=0.717); DBP was 7.3±5.4(AM) and 12.4±8.7 (PM) mmHg (P=0.410); MAP was 4.0±3.1 (AM) and 5.9±4.0 (PM) mmHg (P=0.499).

### CONCLUSIONS

Interarm differences may be present in clinical populations, particularly for DBP. These findings support further investigations into best practices for BP assessments.

Email of Presenting Author: atouihes@msu.edu

## 37

## DOES EARLY ADMINISTRATION OF TRANEXAMIC ACID DECREASE PERIOPERATIVE BLOOD LOSS AND TRANSFUSION RATES IN ADDITION TO INTRAOPERATIVE TRANEXAMIC ACID FOR HIP FRACTURE PATIENTS?

**Jake Stahnke, DO (Resident)**; Shayan Memar (Medical Stu- dent); Amy Meyers, DO (Resident); Matthew Marquart D.O., FAAOS (Faculty); Jacob Lytle, DO (Fellow); Jacob Hinkley, DO, MS (Fellow).

SCS-affiliated Resident Physician or Fellow; Ascension Genesys, MI.

### BACKGROUND

Tranexamic acid (TXA) is an antifibrinolytic agent commonly used in joint replacement surgery with goals to limit postoperative blood loss and decrease risk of transfusion. There continues to be a need for orthopedic research re- volving around TXA use with various orthopedic non-elective procedures. This study aimed to determine whether an early administration of TXA while in the emergency department reduced perioperative blood loss and decreased blood transfusion rates in patients undergoing surgical procedures for hip fractures.

### METHODS

A randomized, placebo-controlled trial included 73 patients who underwent either hemiarthroplasty or closed vs open reduction internal fixation with a cephalo-medullary nail. The treatment group included 37 patients and the control group included 36 patients. The control group only received TXA intraoperatively and postoperatively, while the treatment group received an additional dose of TXA preoperatively.

### RESULTS

The mean age of participants was 80.7 (SD: 11.3) years old, with 51 (70.8%) females and 21 (29.2%) males. The mean time to early administration of TXA for the treatment group was 3.9 (SD: 2.1) hours. The mean surgery length was 1.4 (SD: 0.5) hours for both groups. The preoperative and post- operative hemoglobin levels showed no significant differences between the two groups. No significant association between postoperative transfusion rates between the control group or treatment group was found, p=0.56. The mean amount of intraoperative fluids was 1371.64 cc (SD: 550.2), with no significant difference between the two groups, p=0.37. No significant differences were observed between groups with time to procedure start (p=0.59) or length of surgery (p=0.72). There was 1 (1.4%) patient with a com- plication of deep vein thrombosis in the control group and there were 3 (4.1%) patients who had a complication of pulmonary embolism, two in the control group and one in the treatment group.

### CONCLUSION

No significant differences were seen between treatment group and control group with the early use of TXA in patients undergoing different surgical procedures for hip fractures.

Email of Presenting Author: stahnkejake@gmail.com

## 38

## A PILOT LONGITUDINAL FIXEL-BASED ANALYSIS OF DIFFUSION WEIGHTED MAGNETIC RESONANCE IMAGING DATA: A TRACK TBI STUDY

**Joshua H. Baker, MS (Medical Student)**; Andrew Bender, PhD; Norman Scheel, PhD; Joshua Hubert, BS; Zac Fernandez BS; David C. Zhu, PhD (Faculty); The TRACK TBI Study Investigators.

MSUCOM Medical Student; MSU Department of Radiology.

### BACKGROUND

Constrained spherical deconvolution is a novel modelling technique for diffusion-weighted magnetic resonance imaging (dMRI) data. In combination with a fixel-based analysis (FBA), it specifically addresses the limitations of the diffusion tensor model (DTI), by accounting for complex white matter fiber architecture. Notably, following a FBA pipeline, dMRI data is transformed into fiber bundle elements called “fixels” instead of the standard volume element, a voxel. Therefore, this method may be more sensitive to white matter changes in mild traumatic brain injury (mTBI). In this analysis, we sought to examine the effect of mTBI on the fixel-based metrics fiber density, cross-section, and their product.

### METHODS

Participants were 21 mTBI patients recruited during the TRACK-TBI study, and 18 controls. For analysis, dMRI data acquired at b=1000mm/s2 and b=3000 mm/s2 were concatenated. One Figure represents steps in the FBA pipeline de- scribed by Rafflet and colleagues 2017. An additional step was added, generating a synthetic undistorted b0 image (Schilling et al., 2019, 2020) for susceptibility field distortion correction with “topup” and “eddy”, from the FSL package. Change images were calculated by subtracting timepoint 2 images from timepoint 1 images and dividing by time in years between acquisitions, a method described by (Genc et al., 2018). Whole brain statistical analysis com- paring mTBI patients and controls was performed using the Connectivity-based fixel enhancement (CFE) method (Rafflet et al., 2015), with sex, age, and handedness modeled as confounds. To account for multiple comparisons, non-para- metric permutation testing across whole-brain white-matter fixels was performed.

### RESULTS

Another Figure highlights the neuroanatomical location of positive effects (red-orange) and negative effects (blue) before correction, on the whole-brain tractogram. No significant differences were observed after correction for multiple comparisons.

### CONCLUSION

Clinical imaging of mTBI is challenging, as the etiology of the injury is heterogenous between patients and is difficult to quantify in a real-world sample. This pilot was the first to apply a longitudinal FBA framework to a sub-sample of TRACK-TBI data. We will extend this approach to the entire sample of (n =391) mTBI patients and (n=148) controls. FBA could add to the current understanding of white matter changes following mTBI.

Email of Presenting Author: stahnkejake@gmail.com

## 39

## THE EFFECTS OF ANTERIOR CRUCIATE LIGAMENT RECONSTRUCTION GRAFT TYPE ON PATELLAR TENDON THICKNESS

**Lauren Grasso (Medical Student)**; Matthew Harkey, PhD (Faculty); Lee Chou (Medical Student).

MSUCOM Medical Student; MSUCOM.

Anterior cruciate ligament reconstruction (ACLR) is commonly performed with either a patellar tendon graft or hamstring tendon graft. There is limited research exploring structural outcomes of the patellar tendon after recovery and the long-term effects of ACLR via these auto- grafts. Changes in patellar tendon thickness are related to pain and may be related to inflammation in the tendon. However, it is unclear if patellar tendon thickness differs between patients with patellar tendon or hamstring grafts following ACLR. Therefore, the purpose of this study is to compare patellar tendon thickness between patients with patellar tendon or hamstring grafts following ACLR.

We recruited patients who were 14-35 years old with a primary, unilateral ACLR. During the ultrasound assessment, patients were positioned supine in 30 degrees of knee flexion. A single examiner placed the ultrasound probe longitudinally in line with the patellar tendon with the inferior pole of the patella in view and recorded three ultrasound images. The lead author assessed and averaged patellar tendon thickness across each bilateral ultrasound image using ImageJ. We used independent samples t-tests and Co- hen’s d effect sizes to determine if there were differences in ACLR or contralateral limb patellar tendon thickness be- tween the patients that received patellar tendon and ham- string graft.

We included 26 patients (8=patellar tendon graft, 18=hamstring graft) with an average age of 22.4±6.7 years, weight of 76.3±19.4 kg, height of 173.9±8.4cm, and months since surgery of 19.9±14.9. The patients with a patellar tendon graft (mean±SD: 3.75±0.76mm) had thicker patellar tendons in their ACLR limb than those that received a ham- string graft (mean±SD: 3.27±0.57mm) (t=1.77, p=0.044, d=0.75). However, there was no difference (t=0.67, p=0.26, d=0.28) in contralateral limb patellar tendon thickness be- tween patients with a patellar tendon graft (mean±SD: 3.47±0.38mm) and a hamstring graft (mean±SD: 3.34±0.50mm).

The thicker patellar tendons in patients with a patellar tendon graft post-ACLR may be due to residual swelling and inflammation at the graft site. Future studies need to deter- mine the importance of increased patellar tendon thickness following ACLR and what interventions may be effective at reducing the tendon swelling.

Email of Presenting Author: bakerj77@msu.edu

## 40

## MOTOR CONTROL AND LEARNING OF A TRUNK TRACKING TASK ARE SIMILAR IN INDIVIDUALS WITH AND WITHOUT CHRONIC LOW BACK PAIN

**Lee S. Chou, MPH (Medical Student)**; Nicholas E. Bray; Angela S. Lee, MPH (Faculty); John M. Popovich Jr., PhD, DPT, ATC (Faculty).

MSUCOM Medical Student; MSUCOM.

### BACKGROUND

Individuals with chronic low back pain (CLBP) have deficits in trunk motor control, but the influence of CLBP on motor learning is not well understood. Assessment of motor control and learning in those with CLBP is important for quantifying treatment outcomes and investigating mechanisms of CLBP. Therefore, the purpose of this study was to investigate the effects of CLBP on trunk motor control and learning.

### METHODS

A total of 34 subjects (n=19 with CLBP; n=15 asymptomatic/ no CLBP) participated in this study. Participants with CLBP had symptoms lasting >3 months, while asymptomatic participants had no CLBP lasting more than 3 days over the past years. Exclusion criteria were disorders affecting balance, blindness, neurological disorders, or significant spinal/postural deformity. Participants performed a seated trunk position tracking task that required moving the trunk in the sagittal plane (flexion/extension) to follow a time- varying input signal displayed on a monitor. Trunk position was recorded with string potentiometers, and each trial lasted 30 seconds. Participants performed a total of 20 trunk position tracking trials during each of two laboratory testing sessions (separated by a minimum of 48 hours). Root mean square error (RSME) between the input signal and participants’ output (i.e., trunk position) quantified performance of each trial. Learning was assessed over the acquisition of 20 trials and retention was determined using the average of the first five trials in the second testing session. Statistical comparisons within and between groups were conducted using SPSS^®^(Armonk, NY: IBM Corp), with significance set at p<0.05.

### RESULTS

While both groups demonstrated improvements (reduced RMSE) in performance with successive trials (p<0.05), there was no statistically significant difference in performance between the asymptomatic group versus the CLBP group across the 20 trials during first or second session. There was also no statistically significant difference in retention between the asymptomatic and CLBP groups.

### CONCLUSION

CLBP did not have a significant effect on motor learning as assessed during a trunk-specific motor task. It is possible that individuals with CLBP and asymptotic individuals per- form the task with different movement strategies while maintaining similar performance, however, this would war- rant further investigation.

Email of Presenting Author: choulee@msu.edu

## 41

## MSU AND EMU COLLEGE STUDENT CAREGIVER STUDY

**Nicholas Hallenbeck (Medical Student)**; Sowmyaw Nakkina (Medical Student); Clare Luz, PhD (Faculty).

MSUCOM Medical Student; MSUCOM.

### BACKGROUND

A substantial, growing percentage of U.S. college students are caregivers for an older adult or person living with disabilities. Evidence indicates that College Student Care- givers (CSCs) face physical, mental, social, and financial health challenges that jeopardize their college and career plans. The study aims included understanding their care- giving role, adversities, and advantages of their dual responsibilities, and knowledge and utilization of resources. Findings could help identify CSCs and inform the development of new resources to support them.

### METHODS

A 48-item, electronic, anonymous survey, developed for this IRB approved, exploratory, cross-sectional pilot study was distributed to all MSU and Eastern Michigan University (EMU) students. Students who identified as a CSC, per the definition provided, were included. A gift card opportunity was available. Major primary outcome variables were perceived academic performance and health. Predictor variables included demographics, amount of time and type of caregiving, relationship to care recipient, and perceived support. Frequencies were tabulated on demographics and other categorical variables. Chi-square tests were used to compare different CSC profiles to determine association be- tween variables.

### RESULTS

156 CSCs responded (63% EMU, 37% MSU). The majority were 20-29 years old, female, single, white, undergraduate, and full-time students, providing 1-10 hours/week of care to parents or grandparents with long-term physical conditions. Reported experience with caregiving was not unilaterally positive or negative. Despite clear rewards, respondents reported negative impacts on academics (41%), personal life (30%), and health (27%). 77% voiced concern about delayed educational timelines. Most felt obligated to assume caregiving responsibility and a lack of respite care. Available community/university resources that may have mitigated perceived negative impacts of caregiving were underutilized. Over half reported scholarships and at- tendance/assignment flexibility would help. A third wanted more hybrid/online classes and tuition policies that do not penalize withdrawal.

### CONCLUSIONS

These findings closely align with prior CSC studies and con- firm unique challenges to health, finances, personal life, and academic success. MSU and EMU CSCs identified gaps in services and policy changes that could mitigate negative impacts. Findings will guide resource development and pol- icy recommendations that can be pilot tested in future implementation studies to support student success more adequately.

Email of Presenting Author: nakkinas@msu.edu

## 42

## PERSONALIZED RESEARCH ADVISOR/STUDENT RECOMMENDATION IN MEDICAL EDUCATION USING LARGE LANGUAGE MODELS

**Na Dai (Medical Student)**; Doha Amin; Reshma Dukkipati; Rula Ibrahim; Shweta Bhavsar; Janice Schwartz (Faculty); Carolina Restini (Faculty).

MSUCOM Medical Student; MSUCOM.

### BACKGROUND

Research training is essential in U.S. medical education. Diversity in student backgrounds presents unique challenges in matching research advisors. Research Committees may not have a broad knowledge and access to multiple institutional areas to recommend and direct students to mentors and vice-versa. So, overseeing every niche of the student research background spectrum can be challenging. In addition, advisors may find it difficult to find the best-matched students.

### AIM

We propose a personalized advisor/student recommendation system to generate a list of relevant matches. Hypothesis: Our recommendation system outperforms the baseline of randomly selecting advisors.

### METHODS

350 MSU professors across seven departments are discovered through department faculty Web pages. The hyperlink of each person/lab was extracted, and their home page con- tent was parsed to form their research profile. 219 MSU PI profiles were obtained after data pruning. Textual profiles were sent to OpenAI GPT-3.5 API to achieve their embed- dings, represented by high-dimensional numeric vectors. Those vectors then served as the input to the K-nearest neighbor search (KNN) to recommend the most similar K profiles as matches.

### RESULTS

Recommendation performance based on 10 pseudo- student profiles generated by chatGPT showed that our system outperforms the baseline significantly. Two tailed student t-tests on Precision@3, and 5 rendered p-values 0.0006 and 0.0007. Evaluations on 4 first year MSUCOM students showed that Precision@3 and 5 from our system were 0.50±0.16 and 0.6±0.14, versus 0.33±0.23 and 0.2±0.14 (baseline), with p-value=0.013 on Precision@5. Comparison between MSUCOM and pseudo-student profiles demonstrated the specificity of research description could explain the superiority difference between two experiments.

### CONCLUSION

We proposed a personalized advisor/student recommendation system to support medical student research advisor matching effectively. Results validated our system outperforms the baseline that randomly generates advisor lists. Perspectives: Large language models in industry are computationally demanding, making them impractical to replicate in academia. We built upon their advantage but targeted at challenges in academia. The use of this model can be expected to broaden the research landscape for medical students and promote academic medicine as a career choice.

Email of Presenting Author: daina@msu.edu

## 43

## DEVELOPING COMMUNICATION SKILLS THROUGH COMMUNITY-ENGAGED TEACHING AND LEARNING SERVICES IS ALIGNED WITH HUMANIZED COGNITIVE FACTORS IMPACTING OMS SELF-PERCEPTION.

**Selena Tiberio (Medical Student)**; Carolina Restini, PharmD, PhD; Carrie Nazaroff, PhD; Gabriella Carr (Med- ical Student); David Ibrahim (Medical Student); Sami Abde- laziz (Medical Student); Ryan Matusik, (Medical Student); Elise Torres (Medical Student).

MSUCOM Medical Student; MSUCOM.

### BACKGROUND

Literature data indicate that early knowledge about health-related areas influences Medical Doc- tors’ (MD) educational path decisions. However, no reports investigate the impact of similar strategies implicated by Doctor of Osteopathic Medicine (DO). Thus, we have provided opportunities for pre-clerkship Osteopathic Medical Students (OMS) to deliver knowledge on different health fields and enhance their communication skills with younger generations. We hypothesized that teaching high schoolers about MD and DO professions would enhance cognitive fac- tors impacting OMS self-confidence to acquire and apply humanistic strategies throughout their medical education. We assessed the knowledge and interest of high school students in pursuing medical careers after the interaction with OMS to evaluate verbalized clinical reasoning founded on the central question: “do you know the difference between Human Medicine vs. Osteopathic Medicine (OM)?”

### METHODS

A cross-sectional study evaluated students taking medical career/health-related disciplines in a South- east Michigan public school interacting with the 2nd-year OMS. Anonymous questionnaires about health-related education and osteopathic and allopathic medicine were de- livered before and after the interactions. Statistical analyses: t-test or Chi-square/Person’s correlation (CI95). A theoretical framework of cognition was applied to evaluate OMS self-perception. Independent authors used a constant comparative method to analyze verbatim transcripts from the think-aloud protocols. Utterances were analyzed and grouped into categories and themes.

### RESULTS

Sixty-three high school students (84% female, age average 16.5) participated. There was a 77.8% learning increase about OM (P<.05). There was a 55.6% increase (P<.05) in learning about osteopathic manipulative treatment (OMT). Of the six OMS who were instructors, all felt that the interaction was positive. OMS interpreted learning assessment results and identified increased interest in medicine and an increased understanding of the medical field, OM, and OMT. The most common utterance was related to communication skills, supported by implementing dynamic teaching methods to engage learners. Common themes across OMS responses: receptivity, friendly learning environment, and humanistic purpose to be considered throughout their medical education.

### CONCLUSION

OMS teaching young learners aspiring for pathways in the healthcare professions positively impacted both sides. Community-engaged teaching and learning services represent a contextual factor affecting clinical reasoning, leading to humanized approaches for future patients.

Email of Presenting Author: tiberios@msu.edu

## 44

## THE IMPACT OF PRIOR AUTHORIZATION REMOVAL ON OPIOID USE DISORDER TREATMENT IN MICHIGAN

**Nirmeen Chouaib, BS (Medical Student)**; Marina Haque MD, MPH (Resident); Wael Saasouh, MD (Faculty); Noura Chbeir, MA (Policy Consultant).

MSUCOM Medical Student; MSUCOM.

### INTRODUCTION

Between 2017 to 2022, opioid-related deaths in the United States more than doubled, increasing from 130 deaths per average from overdose to over 294. Additionally, less than 20% of Americans are able to access opioid use disorder (OUD) treatment, with very large disparities in access faced by Medicaid recipients who are much more likely to be prescribed and die from overdose. In December 2019, the state of Michigan chose to eradicate prior authorization, one of the largest barriers to accessing Buprenorphine, a primary medication for treatment. This study sought to analyze barriers to accessing treatment and the impact of Michigan’s prior authorization removal on treatment accessibility.

### METHODS

A mixed methods approach was used. Data stemmed from the following: primary qualitative data from Detroit-area providers, quantitative data from Medicaid reimbursement data for treatment, state opioid misuse related data from 2017-2022, and a comprehensive literature review. A difference-in-difference analysis was conducted between Michigan and Idaho, another state that had not removed prior authorization, to indicate the significance of the removal of prior authorization in Michigan for patients.

### RESULTS

Results showed that between 2019 to 2021 there was an in- crease in both Idaho and Michigan for buprenorphine prescriptions, but Michigan had a 22.8% lower rise in prescriptions than Idaho as well as a 23.5% lower reimbursement rate by Medicaid for prescriptions. Medicaid reimbursement in Idaho was nearly 4x higher for buprenorphine/ naloxone combination in comparison to Michigan. Barriers identified by healthcare systems and providers continued to show that Michigan Medicaid policies restricted access for most Medicaid recipients given its lack of coverage for managed care plan recipients, the patient population Medicaid primarily covers. Other barriers included a significant lack of clinical support for treatment services and lack of awareness about treatment options in Michigan.

### CONCLUSION

Despite the removal of prior authorization for Medicaid recipients, Michigan has a further way to go to help fight the opioid epidemic. Our recommendations would be to base advocacy and policy plans off other states such as Idaho and Colorado which have made it much easier for Medicaid recipients to learn of and afford Buprenorphine.

Email of Presenting Author: chouaibn@msu.edu

## 45

## CARDIAC RELAXATION EFFECTS CAUSED BY THE MYOSIN-SPECIFIC DRUG OMECAMTIV MERCARBIL AND MYOSIN ISOFORM CONTENT

**Benjamin M Cavanaugh (Medical Student)**; Alexandra R Matus; Melissa J Bukowski; Brianna M Schick; Po-Jen Chiang; Donal O’Leary; Joseph T Mannozzi; Charles S Chung, PhD (Faculty).

MSUCOM Medical Student; MSUCOM.

Novel myosin-specific modulator drugs hold promise to improve the lives of patients with cardiac disease. Omecamtiv Mercarbil (OM) is a myosin activator that increased cardiac contractility as well as enhanced relaxation in clinical trials. Recent studies have suggested the rate of cardiac muscle relaxation is positively linked with the rate of mechanical end-systole lengthening. Because this Mechanical Control of Relaxation (MCR) is likely due to myosin detachment and OM directly changes the myosin kinetics, investigating the direct effects of OM on end-systole lengthening may provide insight into the potential treatment of relaxation impaired heart failure with myosin modulators.

In this study, Intact cardiac (multi-cellular) trabecula were isolated from rats, perfused, paced, and treated with OM while relaxation parameters were measured. Because of differences from large animal models, rats were also treated with Propylthiouracil (PTU) for up to 6 weeks prior to dissection to mimic the ratio of beta/alpha myosin heavy chain (MHC) isoforms in humans.

Results indicated the MCR was more pronounced with increasingly higher doses of OM, more than doubling through the physiologic dose range. Altering MHC isoforms enhanced MCR at all OM concentrations with increasing beta/alpha-MHC ratios.

This study has shown myosin modulator drugs have potential to directly affect the MCR of cardiac myocytes, but the effect is likely dependent on MHC isoforms. Targeting myosin pharmacologically may provide a new target for improving cardiac relaxation and diastolic function.

Email of Presenting Author: cavan110@msu.edu

